# The Gut-Liver Axis in Cholestatic Liver Diseases

**DOI:** 10.3390/nu13031018

**Published:** 2021-03-21

**Authors:** Andreas Blesl, Vanessa Stadlbauer

**Affiliations:** 1 Division for Gastroenterology and Hepatology, Department of Internal Medicine, Medical University of Graz, 8036 Graz, Austria; vanessa.stadlbauer@medunigraz.at; 2 Center for Biomarker Research in Medicine (CBmed), 8010 Graz, Austria

**Keywords:** gut-liver axis, primary sclerosing cholangitis, SC-CIP, primary biliary cholangitis, microbiome

## Abstract

The gut-liver axis describes the physiological interplay between the gut and the liver and has important implications for the maintenance of health. Disruptions of this equilibrium are an important factor in the evolution and progression of many liver diseases. The composition of the gut microbiome, the gut barrier, bacterial translocation, and bile acid metabolism are the key features of this cycle. Chronic cholestatic liver diseases include primary sclerosing cholangitis, the generic term secondary sclerosing cholangitis implying the disease secondary sclerosing cholangitis in critically ill patients and primary biliary cirrhosis. Pathophysiology of these diseases is not fully understood but seems to be multifactorial. Knowledge about the alterations of the gut-liver axis influencing the pathogenesis and the outcome of these diseases has considerably increased. Therefore, this review aims to describe the function of the healthy gut-liver axis and to sum up the pathological changes in these cholestatic liver diseases. The review compromises the actual level of knowledge about the gut microbiome (including the mycobiome and the virome), the gut barrier and the consequences of increased gut permeability, the effects of bacterial translocation, and the influence of bile acid composition and pool size in chronic cholestatic liver diseases. Furthermore, therapeutic implications and future scientific objectives are outlined.

## 1. Introduction

Impaired biliary formation or flow can lead to cholestasis [1]. Cholestatic liver diseases can be divided into acute (such as choledocholithiasis and malignancies) and chronic diseases. Chronic cholestatic liver diseases include inherit cholestatic diseases, primary sclerosing cholangitis (PSC), the generic term secondary sclerosing cholangitis (SSC) subsuming diseases caused by mechanical obstruction, ischemia, infections, immune-mediated diseases, or liver damage due to toxic substances, and primary biliary cholangitis (PBC) [2,3]. This review will focus on PSC, SSC in critically ill patients (SC-CIP) as a sub-entity of SSC and PBC because alterations of the gut-liver axis seem to play an important role in the pathogenesis of these diseases and knowledge about alterations of the gut-liver axis may influence future therapeutic strategies.

The occurrence of these diseases is rare, with an incidence between 0.3 and 5.8 per 100,000 people per year [4]. The pathogenesis is still not fully understood, but a multifactorial evolution seems likely. Clinical manifestations can happen in all ages, mainly in the form of elevated cholestasis parameters, jaundice and pruritus. Incidence is not equally distributed between women and men in all three disease types and prognosis without liver transplantation remains limited in many patients [2,4,5].

PSC can affect the intra- and extrahepatic biliary tree. Inflammation of bile ducts leads to progressive liver dysfunction and liver cirrhosis with the potential need for liver transplantation. This disease affects primary men (65–70%) and is associated with inflammatory bowel diseases (IBD) in the majority of patients (70–80%). The risk for hepatobiliary malignancies is increased [6,7,8]. Pharmaceutical treatment is currently not available and mean liver transplantation-free survival is 14.5 years [9,10]. SC-CIP occurs after long-term intensive care treatment with the need for catecholamine treatment and invasive ventilation. Male patients are affected more frequently than women [11]. Prognosis without liver transplantation is often poor and progression to liver cirrhosis can occur within a period of weeks [12,13,14]. PBC leads to progressive destruction of the intrahepatic biliary ducts and occurs most frequently in women of middle age with a sex ratio of 10:1. Clinical symptoms include fatigue, pruritus and jaundice. Due to improved pharmacological treatment options with ursodeoxycholic acid, bezafibrat, and obeticholic acid, liver transplantation is only necessary for 4% of affected patients [2,4,5,15].

The etiologies of these diseases remain widely enigmatic [2]. In PSC, genetic and environmental factors are proposed and associations with the human leukocyte antigen system and other gene loci could be shown, however, a clear causal relationship could not be proven [16,17]. Because recurrence of the disease is possible after liver transplantation, extrahepatic drivers are likely to play a significant role in the pathogenesis [7]. SC-CIP is most likely triggered by ischemic injury of the biliary system during critical illness with consecutive formation of biliary casts and recurrent biliary infections [18]. PBC is a multifactorial disease and environmental triggers like infections (urinary tract infections, infections with mycobacteria, Epstein-Barr virus, chlamydia, *Helicobacter pylori*) and smoking, as well as genetic predisposition and autoimmune factors have been described [5,19,20,21,22,23,24,25].

Recently, data has emerged highlighting the role of the microbiome and the gut barrier in liver diseases, leading to the concept of the gut-liver axis as a common pathogenetic principle and a potential therapeutic target [26]. In this review, we will focus on the interplay between the gut and liver in the evolution and progression of chronic cholestatic liver diseases. We will discuss actual knowledge concentrating on gut permeability, bacterial translocation, bile acids, the gut micro- and mycobiome, and the virome as well as therapeutic implications and future perspectives and research questions.

## 2. The Gut-Liver Axis

**Figure 1:**
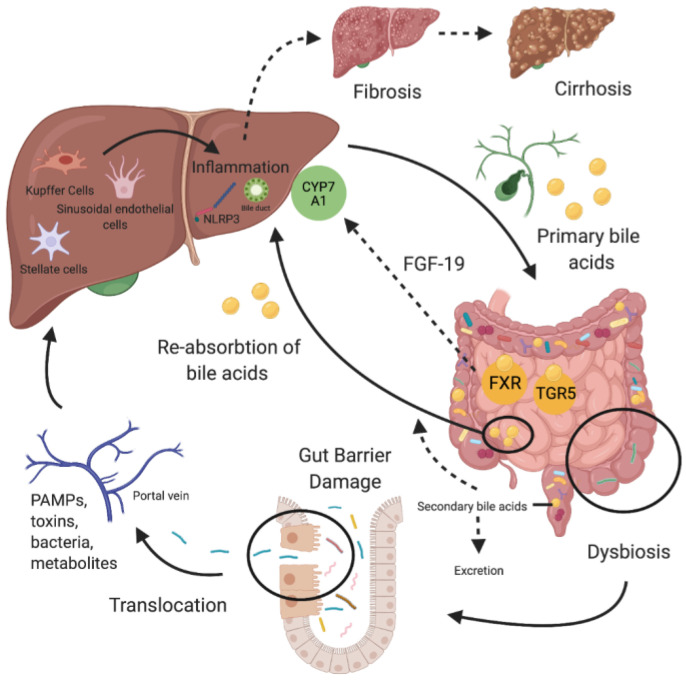
The gut-liver axis in liver diseases. Dysbiosis of the microbiome leads to gut barrier damage enabling translocation of bacteria, toxins, pathogen associated molecular patterns (PAMPs), and metabolites of gut microbes. Via the portal vein, they reach the liver and trigger inflammation cascades leading to fibrosis and cirrhosis in disease courses. Bile acids are synthesized from cholesterol in hepatocytes. After conjugation, they are secreted into the bile ducts and reach the intestine as conjugated primary bile acids. In the gut, bile acids allow digestion of fat and they interact with gut microbes and cellular receptors. Takeda G-protein-coupled receptor 5 (TGR5) and farnesoid X receptor (FXR) are the most important of these receptors. Binding of bile acids to FXR induces formation of fibroblast growth factor 19 (FGF-19) which serves as negative feed-back mechanism for bile acid synthesis in the liver. The majority of bile acids are reabsorbed in the terminal ileum, whereas a small proportion is secreted into the colon, metabolized to secondary bile acids and then also reabsorbed or excreted. Created with BioRender.com. CYP7A1 = Cytochrome P450, Family 7, Subfamily A, Polypeptide 1; NLRP3 = NOD-, LRR- and pyrin domain-containing protein 3.

The communication between the gut and the liver works bidirectionally [27,28,29] (Figure 1). The gut microbiome consists of a thousand species with trillions of microorganisms spanning from bacteria to fungi, protozoa, archaea, and viruses [30]. Composition of the gut microbiome is influenced in early life by mode of birth and breastfeeding, subsequently by age, genetics, food, and drug intake [31,32]. Disturbance of the gut microbiome leads to dysbiosis and further to increased gut permeability allowing translocation of microbes and microbial products named microbial or pathogen-associated molecular patterns (MAMPs/PAMPs) [28,33,34]. Immune receptors of liver cells can identify these patterns once they are in the blood circulation and they can lead to activation of inflammation cascades causing fibrosis and cirrhosis in the course of liver diseases [34]. Vice versa, the liver communicates with the gut through the biliary system and the systemic circulation by releasing bile acids and systemic inflammatory mediators like cytokines [35]. Primary bile acids are synthesized from cholesterol in the liver. Conjugation with taurine or glycine then happens in hepatocytes and the conjugated bile acids are released into the intestine, where they enable the uptake of lipids and fat-soluble vitamins. In the terminal ileum, the majority of bile acids is actively reabsorbed. The remaining 5% are transformed to secondary bile acids by microbial metabolisms in the colon and are passively reabsorbed or excreted. Transformation from primary to secondary bile acids (deconjugation and dihydroxylation) is facilitated by bile salt hydrolases (BSH) and 7α-dehydroxylase expressed by microbes of the gut microbiome including all major phyla (BSH) and the genera *Bacteroides*, *Clostridium*, *Eubacterium*, *Lactobacillus*, and *Escherichia* [34,36,37,38]. Bile acids do not act solely as agents involved in fat digestion, but they additionally work as signaling metabolites that can interact with luminal microbes and cellular receptors of the intestine [37,39,40].

## 3. Gut Permeability and Bacterial Translocation

### 3.1. Introduction

The intestinal barrier is formed by enterocytes which are held together by junctional proteins [41]. A layer of mucins, antibacterial substances, immunoglobulins in the gut lumen and commensal bacteria producing short-chain fatty acids and holding down pathogenic microbes support maintenance of the barrier [34,41,42,43,44]. It regulates transportation of nutrients across tight junctions and prevents microbes from passing the mucosa and entering the systemic circulation [34]. Integrity of the mucosal barrier can be disrupted by dysbiosis of the microbiome caused by inflammation, infections, antibiotic usage, western diet, or alcohol consumption [41,45,46,47,48,49,50,51]. The mucus layer interacts with the microbiome and lower abundance of *Akkermansia muciniphila* was shown to decrease the mucus film [52,53,54,55,56,57]. An impaired gut barrier allows microbes and PAMPs to pass and reach the liver via the portal vein [34]. In the liver they drive inflammation through receptors on Kupffer cells, liver sinusoidal endothelial cells, hepatic stellate cells, and most likely by activating the NLRP3 inflammasome [28,58,59,60,61]. Additionally, short-chain fatty acids like butyrate, choline metabolites involved in accumulation of triglycerides in the liver, and ethanol and its metabolite acetaldehyde are known to play a role in the pathogenesis of liver diseases via the gut [34,62,63,64]. In healthy humans, a certain amount of bacterial translocation is physiological and plays an important role in shaping host immunity. In liver diseases, in particular in cirrhosis, increased bacterial translocation is observed as a pathophysiological mechanism that is influenced by bacterial overgrowth, lymphatic tissue defects, and a mislead response of the immune system to microbes and PAMPs [65,66,67].

Since it is difficult to assess gut permeability and bacterial translocation directly in clinical conditions, biomarkers need to be used. Evaluation of transportation of differently sized sugars across the intestinal epithelium by measuring lactulose, rhamnose, and mannitol urinary excretion after oral ingestion (lactulose:mannitol and lactulose:rhamnose ratios) is considered as the gold standard to characterize the amount of small intestinal permeability in relation to the available absorptive space [68,69,70]. However, the test is challenging to perform for patients, pre-analytic errors can impair the results, and quantitative analytics of different sugars are not broadly available outside research settings [68]. The radioactive 51Cr-EDTA can also be used to screen for increased intestinal permeability by measuring its concentration in urine, however, using radioactive tracers in research settings and also for routine use is difficult [71]. Therefore, biomarkers that can be measured from stool or serum samples are of interest.

Intestinal fatty-acid binding protein (I-FABP) is expressed in enterocytes in the mucosal layer of the jejunum and in small amounts in the colon. Disturbance of epithelial cells leads to distribution of the protein into the systemic circulation and I-FABP plasma levels became a marker of cell death and gut permeability. Associations with intestinal diseases like celiac disease and ischemia have been demonstrated [71,72,73,74,75]. Secretory immunoglobulin A (sIgA) is present in the mucosal surface of the intestine and is involved in keeping gut homeostasis, protects against toxins and pathogens, limits inflammatory reactions, and contributes to the composition of the gut microbiome [44,76,77,78]. Measuring the concentration and composition of sIgA in feces is therefore considered as a suitable parameter to assess the interplay between the immune system and the microbiome at the gut barrier. Zonulin in stool, which has been proposed to be pre-haptoglobin 2, modulates intracellular tight junctions and therefore regulates intestinal permeability [79,80]. It has been proven to be modifiable by microbiome targeted interventions [81,82]. The enzyme diamine oxidase (DAO), liable for oxidative deamination of histamine and present in the intestine from the duodenum to the ileum, inversely correlates with gut permeability of the small intestine when measured in serum and therefore represents a useful permeability marker, which is easier to obtain and store than stool samples [83,84]. Furthermore, tight junction proteins (for example, claudin-3 in urine) and the amino acid citrulline, secreted by enterocytes of the small intestine, can be measured [71]. The PAMP lipopolysaccharide (LPS), which consists of glycolipids from the outer membrane of gram-negative bacteria, is a typical marker for endotoxemia and bacterial translocation. LPS-binding protein (LBP) correlates with LPS and binds to receptor CD14, existing in soluble (sCD14) and in membrane-bound (mCD14) forms, which is involved in triggering inflammation. LBP and sCD14 can be measured with sandwich ELISA kits in plasma and are useful surrogate parameters for endotoxemia [10,85,86]. Calprotectin, a protein located mainly in neutrophils, but also in macrophages and monocytes, is secreted into the intestinal lumen during ongoing inflammation of the intestine and can be measured in feces as a marker of intestinal inflammation [87].

### 3.2. PSC

A connection between gut and liver seems to be obvious in PSC due to the common appearance of IBD in PSC patients and the fact that prior colectomy before liver transplantation decreases the risk of PSC recurrence in the donor liver [88,89]. The IBD-PSC phenotype clinically often presents with an atypical mild right-sided colitis with rectal sparing and backwash ileitis, suggesting a distinct phenotype of the disease [90,91,92]. Increased paracellular permeability, associated with genetic variants of the nucleotide-binding oligomerization domain-containing protein 2 (NOD2), in both CD and UC have been shown [93,94,95,96]. A clear-cut association of genetic variants predisposing to increased gut permeability with the development of PSC however has not been established yet [97]. An important question is whether alterations of permeability are a consequence of the inflammatory reaction or a primary impairment. Data from animal models suggest that disturbance of the gut barrier precedes mucosal inflammation in IBD and that amelioration of the gut barrier leads to reduced inflammation [98,99,100,101,102,103]. Disturbance of the intestinal barrier function has been associated with the development of dysbiosis, which predisposes to colitis in animal models [104]. On the other hand, dysbiosis can alter permeability and cause liver injury. Nakamoto et al. suggested with data from a mouse model that *Klebsiella pneumonia* alters the epithelial barrier in PSC patients and therefore leads to bacterial translocation. Hepatic T-helper 17 cells inducing an immunological response leading to liver injury were correlated with permeability [105]. Additionally, the translocation of *Lactobacillus gasseri* induced interleukin-17–mediated inflammatory injury in the liver of mice [106]. In humans, interferon γ-related biomarkers released from T cells were elevated in PSC and were correlated with transplant-free survival [107]. Abnormal high endotoxin accumulation has been shown in biliary epithelial cells of liver specimens of PSC (and also PBC) patients [108]. Recently, elevation of LBP and sCD14 in a large PSC cohort compared to healthy controls and association of elevation of these markers with reduced transplantation -free survival has been shown. sCD14 was further elevated in patients with hepatobiliary cancer, suggesting a relationship between gut barrier dysfunction, inflammation and cancer development [10]. Also, elevated markers of structural intestinal damage were associated with worse outcome in PSC [109]. A small, earlier conducted study with inclusion of 22 PSC patients could not confirm increased gut permeability with measurement of excretion of lactulose/L-rhamnose, probably due to the small number of included patients and the above described technical challenges of this assay [110]. Taken together, these data implicate that in PSC, increased gut permeability and bacterial translocation are pathophysiological key features. It is still not completely elucidated if gut barrier dysfunction precedes or follows the development of the disease. It can be hypothesized that gut barrier dysfunction and inflammation can perpetuate disease development as part of a “vicious cycle” irrespective of the individual starting point of a patient. Its association with outcome suggests that this is a central feature in PSC and improving gut barrier function seems to be a promising therapeutic target.

### 3.3. SSC

Recently, we were the first to show that biomarkers of gut permeability and bacterial translocation are increased in SC-CIP with elevation of DAO and sCD14 compared to healthy controls. However, we found similar alterations in a group of patients with a history of a critical illness and intensive care treatment without development of SC-CIP [18]. This leaves room for discussion whether these changes are caused by the liver disease itself, by the experienced critical illness, or by an overlap of both. Earlier, increased gut permeability measured by elevation of I-FAB and reduction of citrulline was associated with negative outcome parameters and increased mortality in intensive care patients [111]. Catecholamine treatment leads to disturbance of the intestinal circulation and therefore also alters the gut barrier [112]. Increased gut permeability may then lead to an increase in infectious complications caused by bacterial translocation and may play its part in the development of multiple organ failure [113]. This suggests, that gut barrier dysfunction is a feature of the critical illness and that a “second hit” that yet needs to be defined is necessary to develop SC-CIP. NOD2 polymorphisms, but not polymorphisms in genes associated with cholestatic liver diseases, were more common in SC-CIP [114].

### 3.4. PBC

For PBC, bacterial translocation is also discussed as one aspect of pathogenesis [115]. In BALB/c mice development of histological features comparable to PBC was recognized under long-term exposure to bacterial antigens [116]. In humans, permeability of the stomach and the small intestine was increased in PBC patients [95,117,118]. Lipoteichoic acid, a cell wall component of gram-positive bacteria, was augmented in destroyed bile ducts of liver samples and in serum of PBC patients compared to patients with hepatitis C [119]. LPS levels were higher in PBC than in healthy controls [120]. Together, these findings indicate failure of the gut barrier in PBC patients, but the relevance of this finding in the evolution of the disease remains to be determined.

## 4. Bile Acids

### 4.1. Introduction

Bile acid homeostasis and a functioning enterohepatic circulation are crucial for a healthy gut-liver axis. Accumulation of bile acids can provoke inflammation and oxidative stress in liver cells and leads to hepatotoxicity resulting in biliary fibrosis and cirrhosis [121]. Primary bile acids from the gut lumen are transported into intestinal epithelial cells where they can bind to farnesoid X receptor (FXR) through which they promote transcription of fibroblast growth factor 19 (FGF-19). FGF-19 is secreted into the portal vein and proceeds to the liver and inhibits hepatic bile acid synthesis via CYP7A1 [34,37,121,122,123]. Furthermore, it can interfere with glucose and lipid metabolism [124,125,126,127]. The second important receptor in enterocytes for bile acid binding is Takeda G-protein-coupled receptor 5 (TGR5). It is accountable for glucose, energy, and metabolic homeostasis and promotes anti-inflammatory immune responses [128,129,130,131,132,133]. Bile acids also have direct and indirect influence on the composition of the gut microbiome through the ability to harm bacterial cell membranes and through the promotion of antimicrobial peptides by FXR receptor binding. This influence is stronger in the small intestine and explains the finding of small bowel bacterial overgrowth in PBC, cirrhosis, and in mouse models after bile duct ligation [28,34,134,135,136,137,138]. On the other hand, species like *Escherichia coli* and *Helicobacter* spp. are resistant to bile acids [139,140,141,142], whereas depletion of secondary bile acids, which have the ability to inhibit growth of *Clostridioides difficile* spores, prepares the ground for reduced colonization resistance [143]. As described, secondary bile acids arise from primary bile acids in the colon due to microbial metabolisms. Decreased amounts of secondary bile acids and elevated primary bile acids resulting in a lower secondary/primary bile acid ratio in feces have been linked to dysbiosis in advanced liver cirrhosis [144]. Furthermore, bile acids can harm the intestinal barrier by direct toxicity [123]. Vice versa the gut microbiome, but also antibiotic intake and dietary habits, modulate composition of the bile acid pool and its size, for example by deconjugation of primary bile acids through bile salt hydrolases [134,145]. *Ruminococcus gnavus N53* can produce ursodeoxycholic acid (UDCA), a bile acid considered beneficial for gut health which is used for the treatment of chronic cholestatic liver diseases [146]. Restoration of the bile acid pool with an increase of secondary bile acids was shown after microbiota transplantation in patients with *Clostridioides difficile* infection [147]. Vancomycin intake in obese patients on the other hand led to reduction of alpha diversity of the gut microbiome, limitation of bile acid dehydroxylation, and improvement of peripheral insulin sensitivity [148].

As a consequence of the described bile acid cycle, bile acids can be measured in the bile, in serum, and in stool and therefore awareness of the specimen used is crucial when interpreting bile acid composition data.

### 4.2. PSC

Patients with early-stage PSC and healthy controls had a similar bile acid composition [149], but bile acid pool size in bile fluid was shown to be reduced in PSC and SSC patients, same as it was also observed in patients with obstructive jaundice [150,151,152,153]. Exact mechanisms for this unexpected finding remain widely enigmatic. Reduced hepatocellular secretion of bile acids, leakage of bile acids from the biliary system, and re-absorption are attempted explanations [151]. In a recent large study including more than 500 PSC patients, bile acid levels in plasma and the primary-to-secondary BA ratio were increased in PSC compared to controls and a model including bile acid alterations predicted hepatic decompensation which was associated with an elevation of many bile acids and an increase of conjugated forms [154]. In addition to changes in bile acid composition and pool size, also increased interleukin-8 levels were observed in the bile of PSC patients, proposing ongoing inflammation in disease course [155].

### 4.3. SSC

Only little is known about bile acids in SC-CIP. We could recently demonstrate changes in the serum bile acids profile of patients with SC-CIP including an elevated total serum concentration of bile acids, same as already shown for PBC and PSC [18,156,157]. Furthermore, elevation of conjugated bile acids became evident in SC-CIP and may be caused by a reduction in microbiome diversity and antibiotic use [18,122,158].

### 4.4. PBC

Bile ducts in PBC present a defective biliary HCO3 umbrella, resulting in a reduction of the physiologically alkaline pH close to the surface of cholangiocytes and hepatocytes [159,160]. Taurine conjugates of chenodeoxycholic acid were found to be elevated in the bile fluid of PBC patients. The hypothesis behind this finding is that taurine conjugation protects cholangiocytes from damage due to the failure of the HCO3 umbrella by decreasing hepatotoxicity of hydrophobic bile acids [161]. Also, altered serum and fecal bile acid composition in therapy naïve PBC patients compared to healthy controls were described. PBC patients showed reduced transformation from primary to secondary and from conjugated to unconjugated bile acids. Dysfunctional microbial metabolism leading to failure in dehydroxylation was suggested as a possible mechanism. Furthermore, changes were more pronounced in patients with advanced-stage PBC [162]. In patients suffering from pruritus total and glyco-conjugated primary bile acids in serum were elevated compared to patients without pruritus [163].

### 4.5. Bile Microbiome

Earlier beliefs that bile is a sterile fluid have been refuted [164,165,166]. The existence of a bile microbiome in healthy people was shown for the first time with samples taken during resections of the gallbladder. Same as in the gut lumen, *Firmicutes*, *Bacteroidetes*, *Actinobacteria*, and *Proteobacteria* dominated the bacterial composition [165]. Dependence of the microbial profile from age and sex has been proposed [166].

In PSC patients, decreased alpha diversity and expansion of pathobionts in the bile fluid compared to a healthy cohort was demonstrated. The genera *Enterococcus* and *Staphylococcus* and the species *Enterococcus faecalis* and *Veillonella dispar* showed the strongest increase in PSC patients [150]. *Enterococcus faecalis* is known to cause epithelial barrier disruption and inflammation [167]. In mice, exacerbation of alcoholic liver disease promoted by proton pump inhibitor intake was linked to an increase of *Enterococcus* spp. [168]. *Veillonella dispar* is a pathogen causing joint infections, meningitis, and endocarditis [169,170,171,172,173]. *Veillonella* spp. are increased in patients with Crohn’s disease who suffer recurrent disease after surgical resection [174] and in people with proton pump inhibitor intake [81,175]. There may also be a relation of bile microbiome alterations with disease severity and complications. *Streptococcus* abundance correlated with disease severity and alpha diversity of the bile microbiome only in PSC patients with dysplasia or cholangiocarcinoma. [149]. Furthermore, not only taxonomic composition but also function of the bile microbiome may be altered since an increase of invasive and proinflammatory bacterial metabolic pathways was shown in the bile microbiome of PSC patients [150].

Microbial analysis with conventional culture methods of the bile in SC-CIP during ERCP revealed positive bacterial detection in 95% of all included patients. Approximately 50% of samples showed monomicrobial and the other 50% polymicrobial growth. 50% of microbes found in SC-CIP were gram-positive bacteria with *Enterococcus* spp. being dominant, 30% were gram-negative and in more than 10% of samples candida was found [176]. No sequencing data from bile of patients with SC-CIP and PBC are available to date. Together, findings described in this section demonstrate alteration of the bile acid pool size and bile acid composition in different body compartments (bile, stools, blood) as well as dysbiosis of the bile microbiome and biliary bacterial overgrowth in chronic cholestatic liver diseases. These changes seem to play a significant role in the pathogenesis and progression of chronic cholestatic diseases since outcome parameters could be correlated with these alterations.

## 5. Gut Microbiome

### 5.1. Introduction

Dysbiosis of the gut microbiome, characterized by an imbalance of microbes, as trigger for the evolution and progression of liver diseases is an accepted pathophysiological concept. As pointed out already, dysbiosis influences the gut barrier and bile acid composition and leads to fibrosis and cirrhosis [91,121]. Furthermore, dysbiosis also takes part in the promotion of carcinogenesis of hepatocellular cancer [177]. Alterations of the microbiome have been shown for cholestatic liver diseases, but also for alcohol-induced liver disease (ALD), non-alcoholic fatty liver disease (NAFLD), drug-induced liver injury, viral-induced liver diseases, and cirrhosis [8,27,91,178]. The question always arises when dealing with description of microbiome alterations in various diseases: Are changes in the microbiome the cause or the consequence of the disease? To answer this question not only knowledge of microbial alterations is crucial, but also the ability to understand function and effects of these alterations. In other liver diseases, especially in alcoholic liver disease, this has already been shown: Patients with ALD suffer from bacterial overgrowth and slow bowel transit and present an increase of *Proteobacteria* in favor of *Bacteriodetes* and *Firmicutes*. Elevation of *Bifidobacteteria*, *Streptococci*, and *Enterobacteria* levels has been shown in severe alcoholic hepatitis [91,179,180]. The link to the pathophysiological influence of the gut microbiome on the evolution of ALD is done with studies showing the induction of dysbiosis through alcohol use in animal models and humans without already existing liver diseases [181,182], whereas a single alcohol binge does not seem to induce dysbiosis [183]. Additionally, in patients with alcoholic hepatitis abundance of *Enterococcus faecalis* producing the exotoxin cytolysin was shown to correlate with disease severity and mortality [184]. Findings could not be reproduced for patients with non-alcoholic steatohepatitis [185].

When interpreting microbiome data from different studies one has to keep further in mind that the gut microbiome differs between feces and mucosal biopsies, caused mainly by the different abundance of the genus *Bacteroides* [186]. Additionally, the mucosal microbiome is dependent on sampling methods (for example brushing, biopsy) and, if biopsies were used, from the location biopsies were taken [186,187,188]. On top, the microbiome differs from person to person and is strongly influenced by environmental factors like diet or drug intake [189,190].

### 5.2. PSC

In the last years, characterization of the gut microbiome and determination of differences in PSC, PSC-IBD, IBD, and healthy controls has been of major interest. In a recently published, large, bilateral PSC cohort not only compositional but also functional changes of the microbiome were investigated. PSC patients showed reduced microbial gene richness and different abundance of several species in feces (Table 1). Furthermore, lower abundance of genes involved in vitamin B6 and branched-chain amino acids synthesis could be observed and reduced plasma levels of these metabolites could be correlated with lower liver transplantation-free survival suggesting a connection between disease course and microbiome functions [191]. In another study, PSC patients showed dysbiosis in fecal and salivary probes, independent of the co-existence of IBD in PSC patients. In feces, decrease of alpha diversity (mean species diversity of an ecosystem) using Shannon’s Index and alteration in beta diversity (spatial changes in species composition) were observed. On species level, PSC patients compared to healthy controls showed a decrease of the relative abundance of commensal bacteria and an increase of potentially pathogenic species [192]. In total, we identified 11 peer-reviewed full papers dealing with the microbiome in PSC patients [191,192,193,194,195,196,197,198,199,200,201] (Table 1). These papers present partly heterogenicity in findings, but several similarities can be summed up for the fecal microbiome: PSC patients present lower alpha diversity and dysbiosis compared to healthy controls. Detailed analysis of taxonomic levels is not always consistent, but a few common patterns can be observed: elevation of *Veillonella* and *Clostridium* species and of the genera *Streptococcus*, *Lactobacillus*, and *Enterococcus* and lower abundance of *Faecalibacterium* and *Coprococcus* in PSC. Concomitant IBD seems to have little influence on the microbiome composition in PSC patients. For the mucosal microbiome data is less consistent. In a study using mucosal biopsies and shotgun RNA-sequencing and 16S rRNA analysis the microbial profile and the transcriptome were different in all three groups (PSC, IBD, healthy controls) and PSC-IBD patients showed bile acid signaling pathway upregulation [193]. In two older studies hardly any alterations between the mucosal microbiome in PSC and healthy controls could be observed [200,201].

Reduced alpha diversity is not unique in cholestatic liver diseases, but can also be found in liver diseases of other origins and in multiple other diseases like IBD and metabolic disorders [190,202,203]. Reduced diversity prepares the ground for pathobionts to overgrow commensals and induce diseases. *Clostridioides difficile* infection presents the prototype disease of this mechanism [204]. For PSC, a small pilot trial including ten patients with PSC-IBD could demonstrate improved diversity after fecal microbiome transplantation and a decrease in alkaline phosphatase of more than 50% in 3 patients [205]. *Veillonella* spp. have a significant pathogenic potential [206]. Increased abundance of *Veillonella* was shown to be a distinct biomarker for disease severity in alcoholic and autoimmune hepatitis and cirrhosis [175,206,207,208,209,210]. *Veillonella parvula* is a potential pathogen that is able to produce LPS and induce cytokine release in humans and in vitro and therefore may serve as trigger for induction of hepatic inflammation via the gut-liver axis [206,211]. Furthermore, its abundance was shown to be increased in long-term PPI use and was associated with mortality in cirrhosis [175]. The commensal, butyrate-producing bacterium *Faecalibacterium prausnitzii* is highly abundant in human feces and exerts anti-inflammatory effects through secretion of salicylic acid and inhibition of cytokines activating inflammation cascades [212]. Its reduction was demonstrated earlier in liver diseases like NAFLD, in gastrointestinal disorders like IBD, IBS, and colon cancer, and in metabolic diseases like diabetes mellitus type 2 and obesity as well as in neurologic and psychiatric diseases [212].

In summary, negative alterations of the intestinal microbiome, such as a reduction in diversity with resulting expansion of pathobionts and a loss of beneficial species in PSC have been demonstrated and influence on disease course has been suggested. Together, these findings underline the relationship between the gut and the liver and point out possible pathophysiological mechanisms. However, more work is needed to dissect exactly which factors (disease severity, comorbidities, drug intake, nutrition) cause microbiome changes or are consequences of these factors.

### 5.3. SSC

In SC-CIP, we described the composition of the microbiome in fecal samples [18]. The gut microbiome of SC-CIP patients is characterized by reduced alpha diversity and dysbiosis with increase of the oral inhabitants *Streptococcus parasanguinis* and *Rothia dentocariosa* as well as *Enterococcus faecium.* With the machine learning technique LefSE associations of the genera *Streptococcus*, *Lactobacillus*, *Enterococcus*, *Romboutsia*, and *Butyricicoccus* with SC-CIP could be demonstrated. Additionally, intake of medications (statins, platelet function inhibitors), cirrhosis, BMI, and intestinal inflammation, measured by calprotectin in feces, were found to be potential predictors for microbiome composition. Especially the finding of low alpha diversity and the increase of the genera *Streptococcus*, *Lactobacillus*, and *Enterococcus* overlaps with findings in PSC [192] and other liver diseases [213]. The increase not only of pathobionts, but also of potential beneficial, butyrate-producing bacteria like *Butyricicoccus*, which was demonstrated to be reduced in IBD patients [214,215,216] and the fact that in a mouse model the butyrate-producing strain *Anaerostipes hadrus* has been shown to disturb the gut epithelium [217], demonstrates the complex interplay of the human gut microbiome.

### 5.4. PBC

So far, five studies have investigated the gut microbiome in PBC patients [162,218,219,220,221] (Table 2). The most recent study included 76 PBC patients and compared them to healthy controls. Alpha diversity was reduced in PBC and the increase of the genera *Streptococcus*, *Lactobacillus*, and *Bifidobacterium* in PBC were found to be the main reason for distinct beta diversity between the two groups. Healthy controls showed increase of the class *Clostridia* where the butyrate-producing genera *Faecalibacterium* and *Anaerostipes* belong to. Additionally, the study demonstrated decreased abundance of *Faecalibacterium* in UDCA non-responders compared to responders [218]. Chen et al. evaluated the gut microbiome in UDCA naïve PBC patients and aimed to demonstrate the interplay of the gut microbiome and bile acids as already partly described above. Secondary bile acids in serum correlated positively with enriched genera in the gut of PBC patients and negative with genera enriched in controls, demonstrating that levels of secondary bile acids may be dependent on microbiome composition due to conversion of primary to secondary bile acids by bile salt hydrolases (BSH) and 7α-dehydroxylase through microbes [162,222]. Abe et al. could further demonstrate increased abundance of *Veillonella* in the oral microbiome of patients with autoimmune liver diseases (AIH, PBC) and showed correlation with cytokine levels in saliva [219]. UDCA showed the ability to partly restore the gut microbiome by decreasing the species *Haemophilus* spp., *Streptococcus* spp., and *Pseudomonas* spp. and by increasing *Bacteroidetes* spp., *Sutterella* spp., and *Oscillospira* spp. who were found to be higher in healthy controls [220]. Summing up these findings leads to the conclusion that patterns of microbiome alterations resemble those in PSC and SC-CIP and therefore suggest similar pathophysiological mechanisms concerning the gut liver interplay in those diseases.

Concluding this part of the review, we can summarize that the gut microbiome in chronic cholestatic liver diseases shows decreased diversity, an increase in pathobionts (for example, *Streptococcus*, *Enterococcus*) and a decrease in potentially beneficial, butyrate producing species (for example *Faecalibacterium*). The role of other microbes is less clear. The genus *Lactobacillus*, a very diverse genus with some members that are known to have beneficial effects, has been found to be increased in all three diseases. It is yet unclear if the increased *Lactobacillus species* are pathogenic in cholestatic liver diseases or if they may represent an (insufficient) compensatory mechanism. Furthermore, it remains unknown which of these multiple changes of the composition of the microbiome takes a lead role in disease evolution and which changes are bystanders.

## 6. Gut Mycobiome

### 6.1. Introduction

Although the gut microbiome holds 99% bacteria and fungi represent only a small part of intestinal microbes it would be wrong to consider them irrelevant at all. Fungi only contain 0.01–0.1% of microbial genes [223]. Composition of the intestinal mycobiome seems to be more variable than bacterial composition and very susceptible to environmental influences. Eating habits seem to be a major factor influencing the mycobiome and studies showed different fungal composition dependent on diet [224,225,226]. In studies about the gut mycobiome in total 267 valid species were detected, but only 15 species were described in more than five studies [224,227]. Furthermore, detected fungi are not always capable of inhabiting the gut because of physical matters like body temperature and may be products of dietary or environmental origin. The human gut presents an environment for growth of some *Candida* (for example *Candida albicans*) and *Malassezia* species. *Cladosporium* (including *Aspergillus* and *Penicillium*) and *Dipodascaceae* are considered as having the potential to colonize the gut, although they do not seem to have their origin in the gut. Other species like *Saccharomyces cerevisiae* or many *Aspergillus* species may survive the gut environment, but are reported to be more numerous in the air, soil, or plants. Finally, species like *Debaryomyces hansenii* and *Penicillium aff. commune* are not capable of inhabiting the gut at all [224,228,229]. *Candida*, *Saccharomyces*, *Malassezia*, and *Cladosporium* seem to be the most abundant fungi in the gut and seem to form the core mycobiome [223,230,231]. When investigating the mycobiome one has to be aware of the limitations of analytic methods. Differences in DNA extraction methods, the lack of consensus primer pairs, diverging sequencing techniques, different software packages, and the lack of a complete database for taxonomic assignment make studies different to compare [232,233,234]. Fungi are said to play an important role in the homeostasis of the gut microbiome and in immunity [235,236]. In mice, the ability of *Candida albicans* substitution during antibiotic intake to promote bacterial recovery on the one hand, but to change the gut microbiome composition in the long term on the other hand was demonstrated [237]. The use of antifungal therapy reduced fungal diversity in mice and led to an increase of bacterial pathobionts promoting increased colonic inflammation [238]. Another example of the importance of fungi in keeping the gut healthy is that secretion of certain enzymes by *Saccharomyces boulardii* can neutralize toxins of *Clostridioides difficile* and *Escherichia coli* endotoxins in rats [239,240]. Same to bacteria, fungi also communicate with the immune system through PAMPs [235]. The key receptor to fungi is Dectin-1 (CLEC7A), which detects β-glucans found in the cell wall of fungi and protects against fungal infections [241,242]. Through Dectin-1 fungi can promote production of pro-inflammatory cytokines by caspase-associated recruitment domain-containing protein 9 (CARD9) and induce T helper 17 (Th17) immune responses [243]. In mice, inhibition of Dectin-1 resulted in improvement of colitis and mice with Dectin-1 deficiency were even resistant to dextran sodium sulfate (DSS) and T cell-induced colitis [242]. Dysbiosis of the fungal microbiome and involvement of fungi in the pathogenesis of disease has been demonstrated for IBD, colorectal cancer, obesity, and atherosclerosis [236,244,245,246,247,248,249,250,251], while patients with diabetes mellitus type 1 demonstrated increased fungal diversity [252]. For IBS, fungal dysbiosis was linked to increased intestinal hypersensitivity while the added value of the investigation of the mycobiome for diagnostic or therapeutic aims was doubted in one study [253,254]. Concerning liver diseases, changes of the mycobiome characterized by lower diversity and *Candida* overgrowth for ALD and by lower diversity and distinct fungal composition in NAFLD were demonstrated [235].

### 6.2. PSC

The role of the gut mycobiome in chronic cholestatic liver diseases is not extensively studied yet. A recent work by Lemoinne et al. using ITS2 sequencing could not find differences in alpha diversity comparing PSC, PSC-IBD, IBD, and healthy probands. When taking PSC and PSC-IBD patients together increased diversity in these groups compared to IBD could be demonstrated. Concerning beta diversity, clustering of all four groups could be observed. Additionally, the fungi-to-bacteria diversity ratio was increased in PSC patients. In all patients included in the study, the most abundant phyla were *Ascomycota* and *Basidiomycota.* PSC patients further presented dysbiosis with decrease of *Saccharomyces cerevisiae* species and increased abundance of *Exophiala*, a genus that was only detected in 5 patients [194]. An anti-inflammatory effect of *Saccharomyces cerevisiae*, which is closely related to the probiotic *Saccharomyces boulardii*, was suggested because of the induction of production of the cytokine IL-10 [247]. In mice, *Saccharomyces cerevisiae* ameliorated colitis caused by adherent-invasive *Escherichia coli* [255]. As a reaction to the described results presented by Lemoinne et al. another study group published their mycobiome data of PSC patients in form of a letter [256]. Alpha and beta diversity of the mycobiome were not altered in PSC patients. Whereas general composition of the mycobiome resembled previous findings, the genus *Exophiala* could not be found in any of the 65 PSC patients. *Sordariomycetes*, probably due to the species *Trichocladium griseum*, and the genus *Candida* were increased in PSC patients of this cohort [256]. Regional differences may not explain different findings of these two studies, since both include patients from mainland Europe. Cultivated *Candida albicans* from the bile of PSC patients was shown to induce production of the cytokine interleukin-17A leading to the activation of Th17 cells. Th17 cells protect epithelial and mucosal surfaces from microbes on the one hand, but are involved in the pathogenesis of IBD and other autoimmune diseases on the other hand [257,258,259,260,261,262]. Biliary candida infections have previously been linked to a more severe disease course in PSC patients [263]. Fungal dysbiosis and infections have been linked to liver diseases in case reports: In a single case, systemic Phaeohyphomycosis caused by *Exophiala dermatitidis* induced pruritus, jaundice, abdominal pain, fatique, and fever in a young woman and resembled sclerosing cholangitis on endoscopic retrograde cholangiography (ERC) [264]. Hong et al. presented a case where *Exophiala dermatitidis* was accused to induce liver cirrhosis [265]. The mycobiome has not been investigated for SC-CIP and PBC so far. Taken together, changes in the composition of the mycobiome seem to influence the gut microbiome and may be involved in the pathogenesis of PSC, but the so far available knowledge is limited and partly conflicting. Currently, scientific efforts to investigate the mycobiome and its functions still seem to be at the very beginning.

## 7. Gut Virome

### 7.1. Introduction

Same as fungi, viruses represent under 1% of all microbes inhabiting the gut but absolute numbers range from 10^8^ to 10^9^ virus-like particles (VLP) per gram of stool. The majority of these VLPs are bacteriophages acting as predators for bacteria and influencing the composition of the microbiome and therefore its function [223,266,267,268,269]. The existence of a core gut phageome has been suggested with data from healthy individuals [270]. The second player of the virome are eukaryotic-targeting viruses [223], recently investigated in IBD [271,272]. Directly after birth, the gut becomes exposed to 16 or more families of animal RNA or DNA viruses. [223,273,274]. Zhang et al. found plant-pathogenic RNA viruses to be most abundant in the human gut of healthy probands [275]. Due to conflicting methodology of studies, the need for usage of shotgun metagenomics which imposes a relevant cost factor and demanding bioinformatics and the lack of conserved marker genes, the definition of a healthy virome is not possible yet at all with available data [223,276,277]. Indeed, data point towards an individual but stable and very diverse virome in every human [278]. Composition of the gut virome is dependent on age, geography, ethnicity, urbanization, and diet [279,280,281]. In addition to the interplay between VLPs and the bacterial microbiome, the virome also exerts influence on immune responses [223]. Increase in bacteriophages was shown to alter mucosal immunity and promote intestinal inflammation in mice [282]. Studies investigating VLPs have demonstrated changes of the virome in feces linked to animal viruses found in the gut and higher phage diversity in celiac disease, IBD, and colorectal cancer [283,284,285,286]. Furthermore, alterations of the virome have been described in HIV, graft-versus-host disease, diabetes mellitus type 2, and malnutrition [287,288,289,290]. Pregnant women with diabetes mellitus type 1 had a different composition of the virome as healthy pregnant women [291]. Most data of the virome exist from fecal samples, but studies with mucosal biopsies have recently been investigated for IBD [272,292,293]. Zuo et al. found dysbiosis in IBD patients with increase of *Caudovirales* bacteriophages and was able to correlate dysbiosis with intestinal inflammation and functional impairment [292]. Reduction of *Caudovirales* bacteriophages abundance could be achieved with FMT in patients with *Clostridioides difficile* infection and overgrowth with donor *Caudovirales* was associated with healing [294]. Donor stool with increased diversity of bacteriophages, but with a lower total number of them was more successful in treating *Clostridioides difficile* infections [295]. Interestingly, FMT in graft-versus-host disease led to an increased abundance of *Caudovirales* phages [296]. FMT showed, same as for bacteria, the ability to change the virome of patients towards the composition of the donor [295,297,298,299].

### 7.2. Liver Diseases

The virome has only been investigated in NAFLD, alcoholic hepatitis, and cirrhotic patients [300,301,302]. In NAFLD, 420 different viral species were detected in all patients, but only 19 of them were found in at least 50% of all fecal samples. As control groups healthy individuals and 13 PBC patients with a mild disease course determined by transient elastography were used. Reduced viral diversity and fewer phages in relation to other viruses were detected in NAFLD compared to PBC and healthy probands and changes were further aggravated by the presence of liver fibrosis. In addition, F2-F4 fibrosis was predicted by a model including age, aspartate aminotransferase (AST), and fecal viral diversity with an area under the curve (AUC) of 0.95. Results were not as good when using bacterial diversity [300]. Fecal VLPs showed higher diversity in patients with alcoholic hepatitis and alterations of certain taxa (*Staphylococcus* phages, *Herpesviridae*) could be correlated with disease severity and mortality [301]. In cirrhotic patients on the other hand the link between fecal phages and cirrhosis specifications and 90-day outcome was modest [302]. The virome was not only investigated in feces, but also from plasma. In patients after liver transplantation the plasma virome showed high abundance of *Anelloviruses* which were linked to nephrotoxicity and flares of infections [303]. With exception of the small group of PBC patients included in the NAFLD study by Lang et al. [300], the virome has not been explored for chronic cholestatic liver diseases, but seems to be an important target for investigation.

## 8. Therapeutic Implications

### 8.1. Introduction

**Figure 2:**
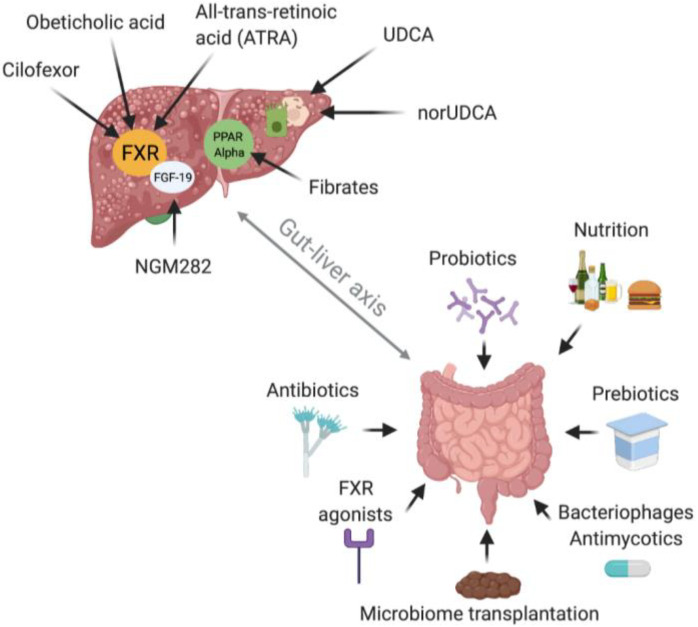
Therapeutic strategies. Current treatment options to modulate the gut microbiome in liver diseases and approved and investigated drugs targeting bile acids for chronic cholestatic liver diseases. Accepted options to therapeutically influence microbiome composition and therefore the gut-liver axis are probiotics, prebiotics, antibiotics, FMT, and nutrition. Antimycotics and Bacteriophages are still in the explorative stage, same as FXR agonists concerning their potential to modulate the gut microbiome. Approved drugs for the treatment of PBC include UDCA, obeticholic acid and fibrates. No approved drugs are available for the treatment of PSC and SC-CIP. NorUDCA, all trans-retinoic acid, cilofexor, other PPAR ligands, and NGM282 are still under investigation. Created with BioRender.com. FXR = Farnesoid X receptor, FGF-19 = Fibroblast growth factor 19, PPAR = Peroxisome proliferator-activated receptor, NGM282 = an engineered fibroblast growth factor 19 analogue, UDCA = Ursodeoxycholic acid, norUDCA = norUrsodeoxycholic acid.

Therapeutic interventions targeting the gut microbiome include prebiotics, probiotics, antibiotics, and fecal microbiome transplantation (FMT) [27] (Figure 2). Prebiotics, which are substrates that are selectively metabolized by beneficial gut bacteria, showed efficacy in preventing liver inflammation and development of steatosis in a mouse model of alcohol-induced hepatic injury and reduced hepatic lipogenesis in humans [304,305,306]. Probiotics as therapeutic options in liver diseases, mainly investigated for ALD, NAFLD, and cirrhosis, have been found to restore gut microbiome composition, to reduce bacterial translocation and to re-establish the gut barrier [27,307,308,309,310,311,312]. Mechanisms reducing liver inflammation include avoidance of TLR4 activation by LPS and inhibition of proinflammatory cytokines [313,314]. The oral use of the antibiotic rifaximin, which is poorly absorbed into the systemic circulation, in patients with liver cirrhosis leads to reduced endotoxemia, modulates the gut microbiome and therefore affects the gut-liver axis [315,316]. FMT proved to be effective in recovering the microbiome and limiting liver inflammation and damage in mice [305,317] and showed reduction of liver disease severity and improved survival after one year in ALD patients [318] and a positive effect on the composition of the microbiome and liver inflammation in patients with NASH [319]. Another important factor influencing microbiome composition which can be used as therapeutic strategy that should not be left unmentioned is nutrition. High fat intake in mice leads to gut dysbiosis with reduction of butyrate-producing microbes and favors the formation of LPS through gram-negative bacteria which leads to liver inflammation and subsequently to NASH [65,320,321]. Results on the impact of dietary interventions and weight loss on gut permeability in liver diseases in humans remain conflicting [322,323]. Further therapeutic strategies to modulate the gut-liver axis include FXR agonists which can treat bile acid dysmetabolism, inhibit bacterial overgrowth, improve gut barrier function and ameliorate liver inflammation, at least in rats [324]. Recently, also therapeutic interventions with bacteriophages have been explored and showed the ability to influence microbiome composition in mice [325]. Bacteriophages targeting *Enterococcus faecalis* lead to reduction of levels of cytolysin, an exotoxin of *Enterococcus faecalis*, in the liver and therefore hinder ethanol-induced liver disease [184]. Concerning the mycobiome, treatment with amphotericin B was successful to prevent alcohol-related liver damage in mice [326].

### 8.2. PSC

In PSC, antibiotic treatment, especially with vancomycin and metronidazole, showed reduction of alkaline phosphatase and bilirubin levels and the Mayo PSC Risk Score. The mechanism is not fully understood and it remains unclear whether this biochemical improvement through antibiotics is caused by direct effects on the microbiome or by immunomodulating effects of vancomycin on TNF-α and TGF-β pathways [327,328,329,330]. Rifaximin was not effective in the treatment of PSC, but minocycline intake over one year led to decrease in alkaline phosphatase levels and the Mayo PSC Risk Score [331,332]. A probiotic formulation of four *Lactobacillus* and two *Bifidobacillus* strains applied over 3 months has not shown efficacy in a small cohort of PSC-IBD patients [333]. In a 13 years old boy suffering from PSC-IBD the combination of a steroid, salazosulfapyridine and a probiotic containing *Lactobacillus casei* led to a notable reduction of cholestasis parameters, but causality with application of the probiotic cannot be drawn from this single case [334]. The pilot study by Allegretti et al. investigating FMT in PSC-IBD patients has been mentioned earlier and indicated restoration of the gut microbiome after FMT and showed the potential to reduce markers of cholestasis [205]. As bile acids are involved in the pathogenesis of PSC, targeting them seems logical. Ursodeoxycholic acid (UDCA) is widely used in PSC, but benefit on long-term clinical outcomes including death is lacking [335]. norUDCA, a derivate of UDCA, and obeticholic acid, a FXR ligand, are currently under investigation for treatment of PSC and showed improvement of alkaline phosphatase levels [336,337]. Further therapeutic strategies being explored are cilofexor, a nonsteroidal FXR agonist; NGM282, an FGF-19 analog, and all-*trans*-retinoic acid (ATRA) which activates FXR [4,338,339,340,341] as well as TGR5 agonists, and inhibitors of the ileal apical sodium bile acid transporter [342].

### 8.3. PBC

The basic therapeutic approach in PBC is to target bile acid metabolism. First-line treatment with UDCA increases bile acid excretion by inducing bile acid flow. Therapeutics with the same approach include norUDCA, obeticholic acid, and fibrates. FXR agonists, FGF-19 analogs, peroxisome proliferator–activated receptor (PPAR) ligands, and apical sodium bile salt transporter (ASBT) inhibitors have the aim to reduce bile acid pool size. Furthermore, modulation of bile acid composition can be achieved with UDCA, norUDCA and PPAR ligands [4,343]. To describe these therapeutic options in detail lies outside the aim of this review. Except for rifampicin for the treatment of pruritus, no data are available for the use of antibiotics, pre- or probiotics or FMT interventions in PBC. Presently ongoing or planned studies targeting the gut microbiome in PSC and PBC are listed in Table 3.

## 9. Future Research Directions and Conclusions

In this review, we have highlighted the role of the gut-liver axis in the chronic cholestatic liver diseases PSC, SC-CIP, and PBC. We have outlined the connection between disturbance of the microbial gut homeostasis, gut barrier defects leading to bacterial translocation and inflammation processes in the liver, and vice versa, the possible modulation of the gut microbiome through bile acids secreted by the liver. Understanding of the function of the gut-liver axis has dramatically improved over the last years, but it remains still a field with a lot of open questions in need of further investigations. Descriptive work of microbiome alterations has reached a satisfactory level, but our understanding of microbiome interactions and function is lagging behind and the complex, multifactorial pathophysiology of chronic cholestatic liver disease is still not fully understood. It seems proven that the gut-liver axis is involved in the evolution and progression of these diseases and therefore presents a promising target for therapeutic interventions. Approved drugs for the treatment of chronic cholestatic liver diseases are still rare and target exclusively bile acids in PBC. Modulation of the gut microbiome has not proven broad efficacy in these diseases yet and no therapeutic options are available in routine use outside clinical trials yet.

For the future, it will be important to increase our understanding of the pathophysiology of these diseases, including the concept of the gut-liver axis and to use this knowledge for the implementation of new treatment strategies. Research aims should focus on the order in which disruptions of the gut-liver axis occur during the evolution of these diseases. To better understand the connection of these disruptions with the pathophysiology of cholestatic liver diseases, translational research efforts would be necessary, starting with in- vitro experiments with anaerobic cultivation of microbiomes and gut barrier models, continuing with animal models and longitudinal observational studies in humans and finally translating these findings into clinical intervention trials with appropriate therapeutic measures. Since chronic cholestatic liver diseases are rare, it is crucial for therapeutic research to establish multinational patient registers to be able to study future drugs in large cohorts with acceptable recruitment timeframes. Further remaining research questions concerning the gut-liver axis in chronic cholestatic liver diseases are listed in Table 4.

## Figures and Tables

**Table 1:** Summary of studies investigating the gut microbiome in PSC. Only full papers are listed. Key findings of microbiome composition are influenced by the personal opinion of the authors of this review because it represents a selection of findings.

Author	Publication Date	Cohort	Sample Origin	Methods	Key Findings of Microbiome Composition in PSC Patients in Feces or Mucosal Biopsies Compared to HC	Assessed Confounders of Microbiome Composition
Kummen et al. [191]	2020	57 PSC, 79 PSC-IBD, 158 HC, 93 IBD	Feces	Metagenomic shotgun sequencing	↓ Diversity ↑ Clostridium ↓ Eubacterium spp. ↓ Ruminococcus obeum	Disease phenotype, UDCA intake, disease duration and severity
Lapidot et al. [192]	2020	17 PSC, 18 PSC-IBD, 30 HC	Feces, saliva	16S rRNA, Illumina MiSeq	↓ Diversity ↑ Streptococcus salivarius ↑ Veillonella dispar ↑ Ruminococcus gnavus ↑ Bacteroides fragilis ↑ Clostridium species ↑ Enterococcus ↑ Blautia ↑ Lactobacillus ↑ Enterobacteriaceae ↓ Bacteroides thetaiotaomicron ↓ Faecalibacterium prausnitzii	Disease phenotype, lifestyle, dietary habits, smoking
Quraishi et al. [193]	2020	10 PSC-IBD, 10 UC, 10 HC	Mucosal biopsies, sigmoid	16S rRNA, Illumina MiSeq	↑ Pseudomonas ↑ Streptoccocus ↑ Haemophilus parainfluenzae ↓ Lachnospiraceae	None
Lemoinne et al. [194]	2020	22 PSC, 27 PSC-IBD, 33 IBD, 30 HC	Feces	16S rRNA, Illumina MiSeq	↓ Diversity ↑ Veillonella ↓ Ruminococcus ↓ Faecalibacterium ↓ Lachnoclostridium ↓ Blautia	Disease phenotype, age, gender, smoking, drug intake, disease severity
Rühlemann et al. [195]	2019	62 PSC, 75 PSC-IBD, 118 UC, 133 HC	Feces	16S rRNA, Illumina MiSeq	↓ Diversity ↑ Veillonella ↑ Streptococcus ↑ Lactobacillus ↑ Enterococcus ↓ Coprococcus ↓ Holdemanella ↓ Desulfovibrio ↓ Faecalibacterium ↓ Clostridium IV	Disease phenotype, UDCA, 5-ASA or azathioprine intake, colonic inflammation, diet
Kummen et al. [196]	2017	30 PSC, 55 PSC-IBD, 36 UC, 263 HC	Feces	16S rRNA, Illumina MiSeq	↓ Diversity ↑ Veillonella ↓ Succinivibrio ↓ Desulfovibrio ↓ Coprococcus ↓ Phascolarctobacterium	Disease phenotype, age, gender, smoking status, BMI, drug intake, liver transplantation, disease duration and severity, concomitant autoimmune diseases
Bajer et al. [197]	2017	11 PSC, 32 PSC-IBD, 32 UC, 31 HC	Feces	16S rRNA, Illumina MiSeq	↑ Rothia ↑ Enterococcus ↑ Streptococcus ↑ Clostridium ↑ Veillonella ↑ Haemophilus ↓ Coprococcus catus ↓ Faecalibacterium prausnitzii ↓ Ruminococcus gnavus ↓ Prevotella copri ↓ Adlercreutzia equolifaciens	Disease phenotype
Sabino et al. [198]	2016	18 PSC, 48 PSC-IBD, 13 UC, 30 CD, 66 HC	Feces	16S rRNA, Illumina MiSeq	↓ Diversity ↑ Streptococcus ↑ Enterococcus ↑ Lactobacillus ↑ Fusobacterium ↑ Morganella ↓ Anaerostipes	Disease phenotype, gender, age, BMI, smoking status, UDCA intake, antibiotic intake, disease severity, liver transplantation
Torres et al. [199]	2016	1 PSC, 19 PSC-IBD, 13 UC, 2 CD, 9 HC	Mucosal biopsies, ileum, right and left Colon	16S rRNA, Illumina MiSeq	↑ Barnesiellaceae ↑ Blautia	Disease phenotype, disease severity
Kevans et al. [200]	2016	31 PSC-IBD, 56 UC	Mucosal biopsies, left colon	16S rRNA, Illumina MiSeq	HC not included in the study	Geographical origin
Rossen et al. [201]	2015	12 PSC-IBD, 11 UC, 9 HC	Mucosal biopsies, ileocoecum	16S rRNA, HITChip	↓ Diversity ↓ uncultured Clostridiales II	None

↓ = lower, ↑ = higher, PSC = primary sclerosing cholangitis, IBD = inflammatory bowel disease, UC = ulcerative colitis, CD = Crohn’s disease, HC = healthy controls.

**Table 2:** Summary of studies investigating the gut microbiome in PBC. Key findings of microbiome composition are influenced by the personal opinion of the authors of this review because it represents a selection of findings.

Author	Publication Date	Cohort	Sample Origin	Methods	Key Findings of Microbiome Composition in PBC Patients in Feces Compared to HC	Assessed Confounders of Microbiome Composition
Furukawa et al. [218]	2020	76 PBC, 23 HC	Feces	16S rRNA, Illumina MiSeq	↓ Diversity ↑ Enterococcus ↑ Streptococcus ↑ Lactobacillus ↑ Bifidobacterium ↓ Clostridiales	UDCA treatment effect, PPI intake
Chen et al. [162]	2020	65 PBC, 109 HC	Feces	16S rRNA, Illumina MiSeq	↑ Lactobacillus ↑ Streptococcus ↑ Klebsiella ↑ Veillonella ↓ Faecalibacterium ↓ Bacteroides	UDCA intake
Abe et al. [219]	2018	39 PBC, 17 AIH, 15 HC	Feces, Saliva	16S rRNA, Terminal restriction fragment length polymorphism	↑ Lactobacillales ↓ Clostridium subcluster XIVa	None
Tang et al. [220]	2018	60 PBC, 80 HC	Feces	16S rRNA, Illumina MiSeq	↓ Diversity ↓ Bacteroidetes spp. ↓ Sutterella ↓Oscillospira ↓ Faecalibacterium ↑ Haemophilus ↑ Veillonella ↑ Clostridium ↑ Lactobacillus ↑ Streptococcus ↑ Pseudomonas ↑ Klebsiella ↑ unknown genus of Enterobacteriaceae	UDCA intake, disease severity, gender, age, BMI
Lv et al. [221]	2016	42 PBC, 30 HC	Feces	16S rRNA, Illumina MiSeq	Multiple alterations mentioned, for example: ↑ Veillonella ↑ Streptococcus ↑ Klebsiella ↑ Enterobacteriaceae ↓ Lachnospiraceae	None

↓ = lower, ↑ = higher, PBC = primary biliary cirrhosis, HC = healthy controls, PPI = proton pump inhibitor, BMI = body mass index.

**Table 3:** Currently ongoing or future studies listed to modulate the gut microbiome in chronic cholestatic liver diseases. Source: clinicaltrails.gov. Search for: primary sclerosing cholangitis, sclerosing cholangitis, primary biliary cholangitis, primary biliary cirrhosis. Shown are studies labeled as recruiting, not yet recruiting, or with unknown status. Studies primary modulating bile acids are not mentioned. Date of access to the website clinicaltrials.gov: 1 February 2021.

Clinical Trials Identifier	Intervention	Disease	Study Design	First Posted	Status	Outcome Measures	Estimated Completion
NCT04678219	Diet (specific carbohydrate diet or a vegan/low-sulfur diet for 8 weeks)	PSC	Open label, randomized	12/20	Recruiting	Shannon Diversity Index, ALP levels	2021
NCT03710122	Vancomycin	PSC	Prospective, randomized, multi-centered, placebo-controlled	10/18	Recruiting	ALP levels (6 to 24 months), elastography	2022
NCT03561584	Sulfasalazine	PSC	Randomized, placebo-controlled	7/18	Recruiting	ALP, AST, ALT, bilirubin, CRP, Mayo PSC risk score, modified fatigue scale, pruritus visual analog scale	2020 (?)
NCT02605213	Vancomycin	PSC (pediatric)	Open label, interventional	5/14	Recruiting	No exact information	2028
NCT00161148	Probiotics (not further defined)	PSC	Randomized, interventional	2005	Unknown	Serum liver tests, pruritus, fatique	2006 (?)
NCT02137668	Vancomycin	PSC	Randomized, interventional	11/15	Unknown	ALP, AST, ALT, GGT, bilirubin, albumin	2016 (?)
NCT03069976	Metronidazole	PSC (pediatric)	Interventional	1/16	Recruiting	AST, ALT, GGT, microbiome composition, bile acid profil	2020 (?)
NCT03521297	Probiotics (Micro V Probiotics)	PBC	Randomized, interventional	1/20	Not yet recruiting	ALP, GGT	2021

PSC = primary sclerosing cholangitis; PBC = primary biliary cholangitis, ALP = alkaline phosphatase, GGT = gamma glutamyl transferase, AST = aspartate aminotransferase, ALT = alanine transaminase, CRP = C-reactive protein, (?) = studies with estimated completion in the past.

**Table 4:** Some future research questions concerning the gut-liver axis in chronic cholestatic liver diseases.

*Are gut barrier defects preceding and therefore triggering cholestatic liver diseases?*
*Are we able to describe longitudinal changes of the microbiome in disease evolution and progression?*
*Can we find enhanced treatment strategies to restore the bile acid composition and pool size in cholestatic liver diseases?*
*Can we identify patients at risk for SC-CIP at intensive care units and implement prophylactic strategies?*
*Is the modulation of the gut microbiome a promising therapeutic target in PSC(-IBD) and PBC?*
*Can we characterize the gut virome in chronic cholestatic liver diseases and can bacteriophages serve as therapeutic option for these diseases?*
*Is the gut mycobiome altered in SC-CIP and PBC and can we find possibilities to modulate the mycobiome for therapeutic aims?*

## Data Availability

The studies reviewed for the manuscript are available from the corresponding author upon reasonable request.

## References

[1] Onofrio F.Q., Hirschfield G.M. (2020). The Pathophysiology of Cholestasis and Its Relevance to Clinical Practice. Clin. Liver Dis..

[2] Hilscher M.B., Kamath P.S., Eaton J.E. (2020). Cholestatic Liver Diseases: A Primer for Generalists and Subspecialists. Mayo. Clin. Proc..

[3] Brooling J., Leal R. (2017). Secondary Sclerosing Cholangitis: A Review of Recent Literature. Curr. Gastroenterol. Rep..

[4] Wagner M., Fickert P. (2020). Drug Therapies for Chronic Cholestatic Liver Diseases. Annu. Rev. Pharmacol. Toxicol..

[5] Tanaka A. (2021). Current understanding of primary biliary cholangitis. Clin. Mol. Hepatol..

[6] Dyson J.K., Beuers U., Jones D.E.J., Lohse A.W., Hudson M. (2018). Primary sclerosing cholangitis. Lancet.

[7] Dean G., Hanauer S., Levitsky J. (2020). The Role of the Intestine in the Pathogenesis of Primary Sclerosing Cholangitis: Evidence and Therapeutic Implications. Hepatology.

[8] LaRusso N.F., Tabibian J.H., O’Hara S.P. (2017). Role of the Intestinal Microbiome in Cholestatic Liver Disease. Dig. Dis..

[9] Gochanour E., Jayasekera C., Kowdley K. (2020). Primary Sclerosing Cholangitis: Epidemiology, Genetics, Diagnosis, and Current Management. Clin. Liver Dis..

[10] Dhillon A.K., Kummen M., Trøseid M., Åkra S., Liaskou E., Moum B., Vesterhus M., Karlsen T.H., Seljeflot I., Hov J.R. (2018). Circulating markers of gut barrier function associated with disease severity in primary sclerosing cholangitis. Liver Int..

[11] Gudnason H.O., Björnsson E.S. (2017). Secondary sclerosing cholangitis in critically ill patients: Current perspectives. Clin. Exp. Gastroenterol..

[12] Kirchner G.I., Rümmele P. (2015). Update on Sclerosing Cholangitis in Critically Ill Patients. Visc. Med..

[13] Kirchner G.I., Scherer M.N., Obed A., Ruemmele P., Wiest R., Froh M., Loss M., Schlitt H.-J., Schölmerich J., Gelbmann C.M. (2010). Outcome of patients with ischemic-like cholangiopathy with secondary sclerosing cholangitis after liver transplantation. Scand. J. Gastroenterol..

[14] Benninger J., Grobholz R., Oeztuerk Y., Antoni C.H., Hahn E.G., Singer M.V., Strauss R. (2005). Sclerosing cholangitis following severe trauma: Description of a remarkable disease entity with emphasis on possible pathophysiologic mechanisms. World J. Gastroenterol..

[15] Selmi C., Bowlus C.L., Gershwin M.E., Coppel R.L. (2011). Primary biliary cirrhosis. Lancet.

[16] Ji S.-G., Juran B.D., Mucha S., Folseraas T., Jostins L., Melum E., Kumasaka N., Atkinson E.J., Schlicht E.M., The UK-PSC Consortium (2017). Genome-wide association study of primary sclerosing cholangitis identifies new risk loci and quantifies the genetic relationship with inflammatory bowel disease. Nat. Genet..

[17] Liu J.Z., Hov J.R., Folseraas T., Ellinghaus E., Rushbrook S.M., Doncheva N.T., Andreassen O.A., Weersma R.K., Weismüller T.J., Eksteen B. (2013). Dense genotyping of immune-related disease regions identifies nine new risk loci for primary sclerosing cholangitis. Nat. Genet..

[18] Blesl A., Jüngst C., Lammert F., Fauler G., Rainer F., Leber B., Feldbacher N., Stromberger S., Wildburger R., Spindelböck W. (2020). Secondary Sclerosing Cholangitis in Critically Ill Patients Alters the Gut–Liver Axis: A Case Control Study. Nutrients.

[19] Tanaka A., Leung P.S., Gershwin M.E. (2019). The genetics of primary biliary cholangitis. Curr. Opin. Gastroenterol..

[20] Corpechot C., Chrétien Y., Chazouillères O., Poupon R. (2010). Demographic, lifestyle, medical and familial factors associated with primary biliary cirrhosis. J. Hepatol..

[21] Burroughs A.K., Rosenstein I.J., Epstein O., Hamilton-Miller J.M., Brumfitt W., Sherlock S. (1984). Bacteriuria and primary biliary cirrhosis. Gut.

[22] Quigley E.M.M. (2016). Primary Biliary Cirrhosis and the Microbiome. Semin. Liver Dis..

[23] Smyk D., Rigopoulou E.I., Zen Y., Abeles R.D., Billinis C., Parés A., Bogdanos D.P. (2012). Role for mycobacterial infection in pathogenesis of primary biliary cirrhosis?. World J. Gastroenterol..

[24] Saadah O.I., Bokhary R.Y. (2013). Anti-mitochondrial antibody positive autoimmune hepatitis triggered by EBV infection in a young girl. Arab. J. Gastroenterol..

[25] Abenavoli L., Arena V., Giancotti F., Vecchio F., Abenavoli S. (2010). Celiac Disease, Primary Biliary Cirrhosis and Helicobacter Pylori Infection: One Link for Three Diseases. Int. J. Immunopathol. Pharmacol..

[26] Kummen M., Hov J.R. (2019). The gut microbial influence on cholestatic liver disease. Liver Int..

[27] Kanmani P., Suganya K., Kim H. (2020). The Gut Microbiota: How Does It Influence the Development and Progression of Liver Diseases. Biomedicines.

[28] Maroni L., Ninfole E., Pinto C., Benedetti A., Marzioni M. (2020). Gut–Liver Axis and Inflammasome Activation in Cholangiocyte Pathophysiology. Cells.

[29] Wang R., Tang R., Li B., Ma X., Schnabl B., Tilg H. (2021). Gut microbiome, liver immunology, and liver diseases. Cell. Mol. Immunol..

[30] Jennison E., Byrne C.D. (2021). The role of the gut microbiome and diet in the pathogenesis of non-alcoholic fatty liver disease. Clin. Mol. Hepatol..

[31] Azad M.B., Konya T., Persaud R.R., Guttman D.S., Chari R.S., Field C.J., Sears M.R., Mandhane P.J., Turvey S.E., Subbarao P. (2015). Impact of maternal intrapartum antibiotics, method of birth and breastfeeding on gut microbiota during the first year of life: A prospective cohort study. BJOG Int. J. Obstet. Gynaecol..

[32] Rinninella E., Raoul P., Cintoni M., Franceschi F., Miggiano G.A.D., Gasbarrini A., Mele M.C. (2019). What is the Healthy Gut Microbiota Composition? A Changing Ecosystem across Age, Environment, Diet, and Diseases. Microorganisms.

[33] Anand G., Zarrinpar A., Loomba R. (2016). Targeting Dysbiosis for the Treatment of Liver Disease. Semin. Liver Dis..

[34] Tripathi A., Debelius J., Brenner D.A., Karin M., Loomba R., Schnabl B., Knight R. (2018). The gut–liver axis and the intersection with the microbiome. Nat. Rev. Gastroenterol. Hepatol..

[35] Stärkel P., Schnabl B. (2016). Bidirectional Communication between Liver and Gut during Alcoholic Liver Disease. Semin. Liver Dis..

[36] Fiorucci S., Carino A., Baldoni M., Santucci L., Costanzi E., Graziosi L., Distrutti E., Biagioli M. (2021). Bile Acid Signaling in Inflammatory Bowel Diseases. Dig. Dis. Sci..

[37] Chen M.L., Takeda K., Sundrud M.S. (2019). Emerging roles of bile acids in mucosal immunity and inflammation. Mucosal Immunol..

[38] Wahlström A., Sayin S.I., Marschall H.-U., Bäckhed F. (2016). Intestinal Crosstalk between Bile Acids and Microbiota and Its Impact on Host Metabolism. Cell Metab..

[39] De Aguiar Vallim T.Q., Tarling E.J., Edwards P.A. (2013). Pleiotropic roles of bile acids in metabolism. Cell Metab..

[40] Lefebvre P., Cariou B., Lien F., Kuipers F., Staels B. (2009). Role of Bile Acids and Bile Acid Receptors in Metabolic Regulation. Physiol. Rev..

[41] Turner J.R. (2009). Intestinal mucosal barrier function in health and disease. Nat. Rev. Immunol..

[42] Gallo R.L., Hooper L.V. (2012). Epithelial antimicrobial defence of the skin and intestine. Nat. Rev. Immunol..

[43] Abreu M.T. (2010). Toll-like receptor signalling in the intestinal epithelium: How bacterial recognition shapes intestinal function. Nat. Rev. Immunol..

[44] Mantis N.J., Rol N., Corthésy B. (2011). Secretory IgA’s complex roles in immunity and mucosal homeostasis in the gut. Mucosal Immunol..

[45] Rao R.K. (2008). Acetaldehyde-induced Barrier Disruption and Paracellular Permeability in Caco-2 Cell Monolayer. Breast Cancer.

[46] Henao-Mejia J., Elinav E., Jin C.-C., Hao L., Mehal W.Z., Strowig T., Thaiss C.A., Kau A.L., Eisenbarth S.C., Jurczak M.J. (2012). Inflammasome-mediated dysbiosis regulates progression of NAFLD and obesity. Nature.

[47] Leclercq S., Cani P.D., Neyrinck A.M., Stärkel P., Jamar F., Mikolajczak M., Delzenne N.M., De Timary P. (2012). Role of intestinal permeability and inflammation in the biological and behavioral control of alcohol-dependent subjects. Brain Behav. Immun..

[48] Martinez-Medina M., Denizot J., Dreux N., Robin F., Billard E., Bonnet R., Darfeuille-Michaud A., Barnich N. (2014). Western diet induces dysbiosis with increased E coli in CEABAC10 mice, alters host barrier function favouring AIEC colonisation. Gut.

[49] Wang Y., Tong J., Chang B., Wang B., Zhang D., Wang B. (2014). Effects of alcohol on intestinal epithelial barrier permeability and expression of tight junction-associated proteins. Mol. Med. Rep..

[50] Tulstrup M.V.-L., Christensen E.G., Carvalho V., Linninge C., Ahrné S., Højberg O., Licht T.R., Bahl M.I. (2015). Antibiotic Treatment Affects Intestinal Permeability and Gut Microbial Composition in Wistar Rats Dependent on Antibiotic Class. PLoS ONE.

[51] Ran Y., Fukui H., Xu X., Wang X., Ebisutani N., Tanaka Y., Maeda A., Makizaki Y., Ohno H., Kondo T. (2020). Alteration of Colonic Mucosal Permeability during Antibiotic-Induced Dysbiosis. Int. J. Mol. Sci..

[52] Everard A., Belzer C., Geurts L., Ouwerkerk J.P., Druart C., Bindels L.B., Guiot Y., Derrien M., Muccioli G.G., Delzenne N.M. (2013). Cross-talk between *Akkermansia muciniphila* and intestinal epithelium controls diet-induced obesity. Proc. Natl. Acad. Sci. USA.

[53] Grander C., Adolph T.E., Wieser V., Lowe P., Wrzosek L., Gyongyosi B., Ward D.V., Grabherr F., Gerner R.R., Pfister A. (2018). Recovery of ethanol-induced *Akkermansia muciniphila* depletion ameliorates alcoholic liver disease. Gut.

[54] Xi M., Li J., Hao G., An X., Song Y., Wei H., Ge W. (2020). Stachyose increases intestinal barrier through *Akkermansia muciniphila* and reduces gut inflammation in germ-free mice after human fecal transplantation. Food Res. Int..

[55] Liu J.-H., Yue T., Luo Z.-W., Cao J., Yan Z.-Q., Jin L., Wan T.-F., Shuai C.-J., Wang Z.-G., Zhou Y. (2020). *Akkermansia muciniphila* promotes type H vessel formation and bone fracture healing by reducing gut permeability and inflammation. Dis. Model. Mech..

[56] Meng X., Wang W., Lan T., Yang W., Yu D., Fang X., Wu H. (2019). A Purified Aspartic Protease from *Akkermansia muciniphila* Plays an Important Role in Degrading Muc2. Int. J. Mol. Sci..

[57] Paone P., Cani P.D. (2020). Mucus barrier, mucins and gut microbiota: The expected slimy partners?. Gut.

[58] Gäbele E., Mühlbauer M., Dorn C., Weiss T.S., Froh M., Schnabl B., Wiest R., Schölmerich J., Obermeier F., Hellerbrand C. (2008). Role of TLR9 in hepatic stellate cells and experimental liver fibrosis. Biochem. Biophys. Res. Commun..

[59] Isayama F., Hines I.N., Kremer M., Milton R.J., Byrd C.L., Perry A.W., McKim S.E., Parsons C., Rippe R.A., Wheeler M.D. (2006). LPS signaling enhances hepatic fibrogenesis caused by experimental cholestasis in mice. Am. J. Physiol. Liver Physiol..

[60] Seki E., De Minicis S., Osterreicher C.H., Kluwe J., Osawa Y., Brenner D.A., Shwabe R.F. (2007). TLR4 enhances TGF-beta signaling and hepatic fibrosis. Nat. Med..

[61] Horst A.K., Neumann K., Diehl L., Tiegs G. (2016). Modulation of liver tolerance by conventional and nonconventional antigen-presenting cells and regulatory immune cells. Cell Mol. Immunol..

[62] Spencer M.D., Hamp T.J., Reid R.W., Fischer L.M., Zeisel S.H., Fodor A.A. (2011). Association between composition of the human gastrointestinal microbiome and development of fatty liver with choline deficiency. Gastroenterology.

[63] Sherriff J.L., O’Sullivan T.A., Properzi C., Oddo J.-L., Adams L.A. (2016). Choline, Its Potential Role in Nonalcoholic Fatty Liver Disease, and the Case for Human and Bacterial Genes. Adv. Nutr..

[64] Chen P., Torralba M., Tan J., Embree M., Zengler K., Stärkel P., Van Pijkeren J.-P., DePew J., Loomba R., Ho S.B. (2015). Supplementation of Saturated Long-Chain Fatty Acids Maintains Intestinal Eubiosis and Reduces Ethanol-induced Liver Injury in Mice. Gastroenterology.

[65] Plaza-Díaz J., Solís-Urra P., Rodríguez-Rodríguez F., Olivares-Arancibia J., Navarro-Oliveros M., Abadía-Molina F., Álvarez-Mercado A. (2020). The Gut Barrier, Intestinal Microbiota, and Liver Disease: Molecular Mechanisms and Strategies to Manage. Int. J. Mol. Sci..

[66] Wiest R., Lawson M., Geuking M. (2014). Pathological bacterial translocation in liver cirrhosis. J. Hepatol..

[67] Matuchansky C. (2014). Bacterial translocation in liver cirrhosis: Site and role in fibrogenesis. J. Hepatol..

[68] Webb D.-L. (2019). Tests of intestinal mucosal hyperpermeability: Many diseases, many biomarkers and a bright future. Best Pract. Res. Clin. Gastroenterol..

[69] Laker M.F., Menzies I.S. (1977). Increase in human intestinal permeability following ingestion of hypertonic solutions. J. Physiol..

[70] Cobden I., Rothwell J., Axon A.T.R. (1981). Passive Permeability in Experimental Intestinal Damage in Rats. Clin. Sci..

[71] Wells J.M., Brummer R.J., Derrien M., Macdonald T.T., Troost F., Cani P.D., Theodorou V., Dekker J., Méheust A., De Vos W.M. (2017). Homeostasis of the gut barrier and potential biomarkers. Am. J. Physiol. Liver Physiol..

[72] Adriaanse M.P.M., Tack G.J., Passos V.L., Damoiseaux J.G.M.C., Schreurs M.W.J., Van Wijck K., Riedl R.G., Masclee A.A.M., Buurman W.A., Mulder C.J.J. (2013). Serum I-FABP as marker for enterocyte damage in coeliac disease and its relation to villous atrophy and circulating autoantibodies. Aliment. Pharmacol. Ther..

[73] Lau E., Marques C., Pestana D., Santoalha M., Carvalho D., Freitas P., Calhau C. (2016). The role of I-FABP as a biomarker of intestinal barrier dysfunction driven by gut microbiota changes in obesity. Nutr. Metab..

[74] Derikx J.P., Schellekens D.H., Acosta S. (2017). Serological markers for human intestinal ischemia: A systematic review. Best Pract. Res. Clin. Gastroenterol..

[75] Chantler S., Griffiths A., Matu J., Davison G., Jones B., Deighton K. (2021). The Effects of Exercise on Indirect Markers of Gut Damage and Permeability: A Systematic Review and Meta-analysis. Sports Med..

[76] Pietrzak B., Tomela K., Olejnik-Schmidt A., Mackiewicz A., Schmidt M. (2020). Secretory IgA in Intestinal Mucosal Secretions as an Adaptive Barrier against Microbial Cells. Int. J. Mol. Sci..

[77] Brandtzaeg P. (2013). Gate-keeper function of the intestinal epithelium. Benef. Microbes.

[78] Yang Y., Palm N.W. (2020). Immunoglobulin A and the microbiome. Curr. Opin. Microbiol..

[79] Fasano A. (2012). Zonulin, regulation of tight junctions, and autoimmune diseases. Ann. N. Y. Acad. Sci..

[80] Tripathi A., Lammers K.M., Goldblum S., Shea-Donohue T., Netzel-Arnett S., Buzza M.S., Antalis T.M., Vogel S.N., Zhao A., Yang S. (2009). Identification of human zonulin, a physiological modulator of tight junctions, as prehaptoglobin-2. Proc. Natl. Acad. Sci. USA.

[81] Horvath A., Leber B., Feldbacher N., Steinwender M., Komarova I., Rainer F., Blesl A., Stadlbauer V. (2020). The effects of a multispecies synbiotic on microbiome-related side effects of long-term proton pump inhibitor use: A pilot study. Sci. Rep..

[82] Horvath A., Leber B., Feldbacher N., Tripolt N., Rainer F., Blesl A., Trieb M., Marsche G., Sourij H., Stadlbauer V. (2020). Effects of a multispecies synbiotic on glucose metabolism, lipid marker, gut microbiome composition, gut permeability, and quality of life in diabesity: A randomized, double-blind, placebo-controlled pilot study. Eur. J. Nutr..

[83] Comas-Basté O., Sánchez-Pérez S., Veciana-Nogués M.T., Latorre-Moratalla M., Vidal-Carou M.D.C. (2020). Histamine Intolerance: The Current State of the Art. Biomolecules.

[84] Honzawa Y., Nakase H., Matsuura M., Chiba T. (2011). Clinical significance of serum diamine oxidase activity in inflammatory bowel disease: Importance of evaluation of small intestinal permeability. Inflamm. Bowel Dis..

[85] Laugerette F., Vors C., Alligier M., Pineau G., Drai J., Knibbe C., Morio B., Lambert-Porcheron S., Laville M., Vidal H. (2020). Postprandial Endotoxin Transporters LBP and sCD14 Differ in Obese vs. Overweight and Normal Weight Men during Fat-Rich Meal Digestion. Nutrients.

[86] Tamaki S., Kanazawa A., Sato J., Tamura Y., Asahara T., Takahashi T., Matsumoto S., Yamashiro Y., Watada H. (2019). Clinical factors associated with bacterial translocation in Japanese patients with type 2 diabetes: A retrospective study. PLoS ONE.

[87] Liu F., Lee S.A., Riordan S.M., Zhang L., Zhu L. (2020). Global Studies of Using Fecal Biomarkers in Predicting Relapse in Inflammatory Bowel Disease. Front. Med..

[88] Alabraba E., Nightingale P., Gunson B., Hubscher S., Olliff S., Mirza D., Neuberger J. (2009). A re-evaluation of the risk factors for the recurrence of primary sclerosing cholangitis in liver allografts. Liver Transplant..

[89] Vera A., Moledina S., Gunson B., Hubscher S., Mirza D., Olliff S., Neuberger J. (2002). Risk factors for recurrence of primary sclerosing cholangitis of liver allograft. Lancet.

[90] Loftus E.V., Harewood G.C., Loftus C.G., Tremaine W.J., Harmsen W.S., Zinsmeister A.R., Jewell D., Sandborn W.J. (2005). PSC-IBD: A unique form of inflammatory bowel disease associated with primary sclerosing cholangitis. Gut.

[91] Chopyk D.M., Grakoui A. (2020). Contribution of the Intestinal Microbiome and Gut Barrier to Hepatic Disorders. Gastroenterology.

[92] Aranake-Chrisinger J., Dassopoulos T., Yan Y., Nalbantoglu I. (2020). Primary sclerosing cholangitis associated colitis: Characterization of clinical, histologic features, and their associations with liver transplantation. World J. Gastroenterol..

[93] Michielan A., D’Incà R. (2015). Intestinal Permeability in Inflammatory Bowel Disease: Pathogenesis, Clinical Evaluation, and Therapy of Leaky Gut. Mediat. Inflamm..

[94] Salim S.Y., Söderholm J.D. (2011). Importance of disrupted intestinal barrier in inflammatory bowel diseases. Inflamm. Bowel Dis..

[95] Fukui H. (2016). Increased Intestinal Permeability and Decreased Barrier Function: Does It Really Influence the Risk of Inflammation?. Inflamm. Intest. Dis..

[96] Buhner S., Buning C., Genschel J., Kling K., Herrmann D., Dignass A., Kuechler I., Krueger S., Schmidt H.H.-J., Lochs H. (2006). Genetic basis for increased intestinal permeability in families with Crohn’s disease: Role of CARD15 3020insC mutation?. Gut.

[97] Karlsen T.H., Hampe J., Wiencke K., Schrumpf E., Thorsby E., Lie B.A., Broomé U., Schreiber S., Boberg K.M. (2007). Genetic Polymorphisms Associated With Inflammatory Bowel Disease Do Not Confer Risk for Primary Sclerosing Cholangitis. Am. J. Gastroenterol..

[98] Yu L.C.-H. (2018). Microbiota dysbiosis and barrier dysfunction in inflammatory bowel disease and colorectal cancers: Exploring a common ground hypothesis. J. Biomed. Sci..

[99] Madsen K.L., Malfair D., Gray D., Doyle J.S., Jewell L.D., Fedorak R.N. (1999). Interleukin-10 gene-deficient mice develop a primary intestinal permeability defect in response to enteric microflora. Inflamm. Bowel Dis..

[100] Olson T.S., Reuter B.K., Scott K.G.-E., Morris M.A., Wang X.-M., Hancock L.N., Burcin T.L., Cohn S.M., Ernst P.B., Cominelli F. (2006). The primary defect in experimental ileitis originates from a nonhematopoietic source. J. Exp. Med..

[101] Nenci A., Becker C., Wullaert A., Gareus R., Van Loo G., Danese S., Huth M., Nikolaev A., Neufert C., Madison B. (2007). Epithelial NEMO links innate immunity to chronic intestinal inflammation. Nature.

[102] Nighot P., Al-Sadi R., Rawat M., Guo S., Watterson D.M., Ma T. (2015). Matrix metalloproteinase 9-induced increase in intestinal epithelial tight junction permeability contributes to the severity of experimental DSS colitis. Am. J. Physiol. Liver Physiol..

[103] Liu X., Xu J., Mei Q., Han L., Huang J. (2012). Myosin Light Chain Kinase Inhibitor Inhibits Dextran Sulfate Sodium-Induced Colitis in Mice. Dig. Dis. Sci..

[104] Couturier-Maillard A., Secher T., Rehman A., Normand S., De Arcangelis A., Haesler R., Huot L., Grandjean T., Bressenot A., Delanoye-Crespin A. (2013). NOD2-mediated dysbiosis pre-disposes mice to transmissible colitis and colorectal cancer. J. Clin. Investig..

[105] Nakamoto N., Sasaki N., Aoki R., Miyamoto K., Suda W., Teratani T., Suzuki T., Koda Y., Chu P.-S., Taniki N. (2019). Gut pathobionts underlie intestinal barrier dysfunction and liver T helper 17 cell immune response in primary sclerosing cholangitis. Nat. Microbiol..

[106] Tedesco D., Thapa M., Chin C.Y., Ge Y., Gong M., Li J., Gumber S., Speck P., Elrod E.J., Burd E.M. (2018). Alterations in Intestinal Microbiota Lead to Production of Interleukin 17 by Intrahepatic γδ T-Cell Receptor–Positive Cells and Pathogenesis of Cholestatic Liver Disease. Gastroenterology.

[107] Dhillon A.K., Rupp C., Bergquist A., Voitl R., Folseraas T., Trøseid M., Midttun Ø., Ueland P.M., Karlsen T.H., Vesterhus M. (2021). Associations of neopterin and kynurenine–tryptophan ratio with survival in primary sclerosing cholangitis. Scand. J. Gastroenterol..

[108] Sasatomi K., Noguchi K., Sakisaka S., Sata M., Tanikawa K. (1998). Abnormal accumulation of endotoxin in biliary epithelial cells in primary biliary cirrhosis and primary sclerosing cholangitis. J. Hepatol..

[109] Tornai T., Palyu E., Vitalis Z., Tornai I., Tornai D., Antal-Szalmas P., Norman G.L., Shums Z., Veres G., Dezsofi A. (2017). Gut barrier failure biomarkers are associated with poor disease outcome in patients with primary sclerosing cholangitis. World J. Gastroenterol..

[110] Björnsson E., Cederborg A., Åkvist A., Simren M., Stotzer P.-O., Bjarnason I. (2005). Intestinal permeability and bacterial growth of the small bowel in patients with primary sclerosing cholangitis. Scand. J. Gastroenterol..

[111] Piton G., Belon F., Cypriani B., Regnard J., Puyraveau M., Manzon C., Navellou J.-C., Capellier G. (2013). Enterocyte Damage in Critically Ill Patients Is Associated With Shock Condition and 28-Day Mortality. Crit. Care Med..

[112] Jung C.Y., Bae J.M. (2021). Pathophysiology and protective approaches of gut injury in critical illness. Yeungnam Univ. J. Med..

[113] Deitch E.A. (2012). Gut-origin sepsis: Evolution of a concept. Surgeon.

[114] Jüngst C., Stadlbauer V., Reichert M.C., Zimmer V., Weber S.N., Ofner-Ziegenfuß L., Voigtländer T., Spindelböck W., Fickert P., Kirchner G.I. (2017). NOD2 gene variants confer risk for secondary sclerosing cholangitis in critically ill patients. Sci. Rep..

[115] Giordano D.M., Pinto C., Maroni L., Benedetti A., Marzioni M. (2018). Inflammation and the Gut-Liver Axis in the Pathophysiology of Cholangiopathies. Int. J. Mol. Sci..

[116] Haruta I., Kikuchi K., Hashimoto E., Nakamura M., Miyakawa H., Hirota K., Shibata N., Kato H., Arimura Y., Kato Y. (2010). Long-term bacterial exposure can trigger nonsuppurative destructive cholangitis associated with multifocal epithelial inflammation. Lab. Investig..

[117] Feld J.J., Meddings J., Heathcote E.J. (2006). Abnormal Intestinal Permeability in Primary Biliary Cirrhosis. Dig. Dis. Sci..

[118] Di Leo V., Venturi C., Baragiotta A., Martines D., Floreani A. (2003). Gastroduodenal and intestinal permeability in primary biliary cirrhosis. Eur. J. Gastroenterol. Hepatol..

[119] Haruta I., Hashimoto E., Kato Y., Kikuchi K., Kato H., Yagi J., Uchiyama T., Kobayash M., Shiratori K. (2006). Lipoteichoic acid may affect the pathogenesis of bile duct damage in primary biliary cirrhosis. Autoimmunity.

[120] Zhao J., Zhao S., Zhou G., Liang L., Guo X., Mao P., Zhou X., Wang H., Nan Y., Xu D. (2011). Altered biliary epithelial cell and monocyte responses to lipopolysac-charide as a TLR ligand in patients with primary biliary cirrhosis. Scand. J. Gastroenterol..

[121] Yang T., Khan G.J., Wu Z., Wang X., Zhang L., Jiang Z. (2019). Bile acid homeostasis paradigm and its connotation with cholestatic liver diseases. Drug Discov. Today.

[122] Abenavoli L., Procopio A.C., Fagoonee S., Pellicano R., Carbone M., Luzza F., Invernizzi P. (2020). Primary Biliary Cholangitis and Bile Acid Farnesoid X Receptor Agonists. Diseases.

[123] Yang H., Duan Z. (2016). Bile Acids and the Potential Role in Primary Biliary Cirrhosis. Digestion.

[124] Copple B.L., Li T. (2016). Pharmacology of bile acid receptors: Evolution of bile acids from simple detergents to complex signaling molecules. Pharmacol. Res..

[125] Sinal C.J., Tohkin M., Miyata M., Ward J.M., Lambert G., Gonzalez F.J. (2000). Targeted Disruption of the Nuclear Receptor FXR/BAR Impairs Bile Acid and Lipid Homeostasis. Cell.

[126] Zou B., Yang W., Tang Y., Hou Y., Tang T., Qu S. (2020). Intestinal microbiota-farnesoid X receptor axis in metabolic diseases. Clin. Chim. Acta.

[127] Chiang J.Y.L., Ferrell J.M. (2020). Bile acid receptors FXR and TGR5 signaling in fatty liver diseases and therapy. Am. J. Physiol. Liver Physiol..

[128] Schaap F.G., Trauner M., Jansen P.L.M. (2014). Bile acid receptors as targets for drug development. Nat. Rev. Gastroenterol. Hepatol..

[129] Thomas C., Gioiello A., Noriega L., Strehle A., Oury J., Rizzo G., Macchiarulo A., Yamamoto H., Mataki C., Pruzanski M. (2009). TGR5-Mediated Bile Acid Sensing Controls Glucose Homeostasis. Cell Metab..

[130] Perino A., Schoonjans K. (2015). TGR5 and Immunometabolism: Insights from Physiology and Pharmacology. Trends Pharmacol. Sci..

[131] Holter M.M., Chirikjian M.K., Govani V.N., Cummings B.P. (2020). TGR5 Signaling in Hepatic Metabolic Health. Nutrients.

[132] Ding J.H., Jin Z., Yang X.X., Lou J., Shan W.X., Hu Y.X., Du Q., Liao Q.-S., Xie R., Xu J.-Y. (2020). Role of gut microbiota via the gut-liver-brain axis in digestive diseases. World J. Gastroenterol..

[133] Gulamhusein A.F., Hirschfield G.M. (2019). Primary biliary cholangitis: Pathogenesis and therapeutic opportunities. Nat. Rev. Gastroenterol. Hepatol..

[134] Begley M., Gahan C.G., Hill C. (2005). The interaction between bacteria and bile. FEMS Microbiol. Rev..

[135] Liu Chen Kiow J., Vincent C., Sidani S., Bouin M. (2019). High occurrence of small intestinal bacterial overgrowth in primary biliary cholangitis. Neurogastroenterol. Motil..

[136] Bauer T.M., Steinbrückner B., Brinkmann F.E., Ditzen A.K., Schwacha H., Aponte J.J., Pelz K., Kist M., Blum H. (2001). Small intestinal bacterial overgrowth in patients with cirrhosis: Prevalence and relation with spontaneous bacterial peritonitis. Am. J. Gastroenterol..

[137] Fouts D.E., Torralba M., Nelson K.E., Brenner D.A., Schnabl B. (2012). Bacterial translocation and changes in the intestinal microbiome in mouse models of liver disease. J. Hepatol..

[138] Inagaki T., Moschetta A., Lee Y.-K., Peng L., Zhao G., Downes M., Yu R.T., Shelton J.M., Richardson J.A., Repa J.J. (2006). Regulation of antibacterial defense in the small intestine by the nuclear bile acid receptor. Proc. Natl. Acad. Sci. USA.

[139] Carpenter H.A. (1998). Bacterial and parasitic cholangitis. Mayo. Clin. Proc..

[140] Brook I. (1989). Aerobic and anaerobic microbiology of biliary tract disease. J. Clin. Microbiol..

[141] Gänzle M.G., Hertel C., van der Vossen J.M., Hammes W.P. (1999). Effect of bacteriocin-producing lactobacilli on the survival of *Escherichia coli* and *Listeria* in a dynamic model of the stomach and the small intestine. Int. J. Food Microbiol..

[142] Hirai Y. (1999). The interaction of bile acids and *Helicobacter pylori*. J. Gastroenterol..

[143] Theriot C.M., Bowman A.A., Young V.B. (2016). Antibiotic-Induced Alterations of the Gut Microbiota Alter Secondary Bile Acid Production and Allow for Clostridium difficile Spore Germination and Outgrowth in the Large Intestine. mSphere.

[144] Kakiyama G., Pandak W.M., Gillevet P.M., Hylemon P.B., Heuman D.M., Daita K., Takei H., Muto A., Nittono H., Ridlon J.M. (2013). Modulation of the fecal bile acid profile by gut microbiota in cirrhosis. J. Hepatol..

[145] Ridlon J.M., Kang D.J., Hylemon P.B., Bajaj J.S. (2014). Bile acids and the gut microbiome. Curr. Opin. Gastroenterol..

[146] Lee J.Y., Arai H., Nakamura Y., Fukiya S., Wada M., Yokota A. (2013). Contribution of the 7β-hydroxysteroid dehydrogenase from *Ruminococcus gnavus* N53 to ursodeoxycholic acid formation in the human colon. J. Lipid. Res..

[147] Weingarden A.R., Chen C., Bobr A., Yao D., Lu Y., Nelson V.M., Sadowski M.J., Khoruts A. (2014). Microbiota transplantation restores normal fecal bile acid composition in recurrent *Clostridium* difficile infection. Am. J. Physiol. Gastrointest. Liver Physiol..

[148] Vrieze A., Out C., Fuentes S., Jonker L., Reuling I., Kootte R.S., van Nood E., Holleman F., Knaapen M., Romijn J.A. (2014). Impact of oral vancomycin on gut microbiota, bile acid metabolism, and insulin sensitivity. J. Hepatol..

[149] Pereira P., Aho V., Arola J., Boyd S., Jokelainen K., Paulin L., Auvinen P., Färkkilä M. (2017). Bile microbiota in primary sclerosing cholangitis: Impact on disease progression and development of biliary dysplasia. PLoS ONE.

[150] Liwinski T., Zenouzi R., John C., Ehlken H., Rühlemann M.C., Bang C., Groth S., Lieb W., Kantowski M., Andersen N. (2020). Alterations of the bile microbiome in primary sclerosing cholangitis. Gut.

[151] Fickert P., Wagner M. (2017). Biliary bile acids in hepatobiliary injury—What is the link?. J. Hepatol..

[152] Woolbright B.L., Dorko K., Antoine D.J., Clarke J.I., Gholami P., Li F., Kumer S.C., Schmitt T.M., Forster J., Fan F. (2015). Bile acid-induced necrosis in primary human hepatocytes and in patients with obstructive cholestasis. Toxicol. Appl. Pharmacol..

[153] Gauss A., Ehehalt R., Lehmann W.D., Erben G., Weiss K.H., Schaefer Y. (2013). Biliary phosphatidylcholine and lysophosphatidylcholine profiles in sclerosing cholangitis. World J. Gastroenterol..

[154] Mousa O.Y., Juran B.D., McCauley B.M., Vesterhus M.N., Folseraas T., Turgeon C.T., Ali A.H., Schlicht E.M., Atkinson E.J., Hu C. (2020). Bile Acid Profiles in Primary Sclerosing Cholangitis and their Ability to Predict Hepatic Decompensation. Hepatology.

[155] Zweers S.J., Shiryaev A., Komuta M., Vesterhus M., Hov J.R., Perugorria M.J., De Waart D.R., Chang J.-C., Tol S., Velde A.A.T. (2016). Elevated interleukin-8 in bile of patients with primary sclerosing cholangitis. Liver Int..

[156] Mouzaki M., Wang A.Y., Bandsma R., Comelli E.M., Arendt B.M., Zhang L., Fung S., Fischer S.E., McGilvray I.G., Allard J.P. (2016). Bile Acids and Dysbiosis in Non-Alcoholic Fatty Liver Disease. PLoS ONE.

[157] Trottier J., Białek A., Caron P., Straka R.J., Heathcote J., Milkiewicz P., Barbier O. (2012). Metabolomic profiling of 17 bile acids in serum from patients with primary biliary cirrhosis and primary sclerosing cholangitis: A pilot study. Dig. Liver Dis..

[158] Devkota S., Wang Y., Musch M.W., Leone V., Fehlner-Peach H., Nadimpalli A., Jabri B. (2012). Dietary-fat-induced taurocholic acid promotes pathobiont expansion and colitis in Il10-/- mice. Nature.

[159] Beuers U., Hohenester S., Wenniger L.J.M.D.B., Kremer A.E., Jansen P.L.M., Elferink R.P.J.O. (2010). The biliary HCO3− umbrella: A unifying hypothesis on pathogenetic and therapeutic aspects of fibrosing cholangiopathies. Hepatology.

[160] Hohenester S., Wenniger L.M.D.B., Paulusma C.C., Van Vliet S.J., Jefferson D.M., Elferink R.P.O., Beuers U. (2011). A biliary HCO3−umbrella constitutes a protective mechanism against bile acid-induced injury in human cholangiocytes. Hepatology.

[161] Dilger K., Hohenester S., Winkler-Budenhofer U., Bastiaansen B.A., Schaap F.G., Rust C., Beuers U. (2012). Effect of ursodeoxycholic acid on bile acid profiles and intestinal detoxification machinery in primary biliary cirrhosis and health. J. Hepatol..

[162] Chen W., Wei Y., Xiong A., Li Y., Guan H., Wang Q., Miao Q., Bian Z., Xiao X., Lian M. (2019). Comprehensive Analysis of Serum and Fecal Bile Acid Profiles and Interaction with Gut Microbiota in Primary Biliary Cholangitis. Clin. Rev. Allergy Immunol..

[163] Hegade V.S., Pechlivanis A., McDonald J.A.K., Rees D., Corrigan M., Hirschfield G.M., Taylor-Robinson S.D., Holmes E., Marchesi J.R., Kendrick S. (2019). Autotaxin, bile acid profile and effect of ileal bile acid transporter inhibition in primary biliary cholangitis patients with pruritus. Liver Int..

[164] Tajeddin E., Sherafat S.J., Majidi M.R.S., Alebouyeh M., Alizadeh A.H.M., Zali M.R. (2016). Association of diverse bacterial communities in human bile samples with biliary tract disorders: A survey using culture and polymerase chain reaction-denaturing gradient gel electrophoresis methods. Eur. J. Clin. Microbiol. Infect. Dis..

[165] Molinero N., Ruiz L., Milani C., Gutiérrez-Díaz I., Sánchez B., Mangifesta M., Segura J., Cambero I., Campelo A.B., García-Bernardo C.M. (2019). The human gallbladder microbiome is related to the physiological state and the biliary metabolic profile. Microbiome.

[166] Serra N., Di Carlo P., D’Arpa F., Battaglia E., Fasciana T., Gulotta G., Maida C.M., Rodolico V., Giammanco A., Sergi C. (2021). Human bile microbiota: A retrospective study focusing on age and gender. J. Infect. Public Health.

[167] Steck N., Hoffmann M., Sava I.G., Kim S.C., Hahne H., Tonkonogy S.L., Mair K., Krueger D., Pruteanu M., Shanahan F. (2011). Enterococcus faecalis Metalloprotease Compromises Epithelial Barrier and Contributes to Intestinal Inflammation. Gastroenterology.

[168] Llorente C., Jepsen P., Inamine T., Wang L., Bluemel S., Wang H.J., Bluemel S., Wang H.J., Loomba R., Bajaj J.S. (2017). Gastric acid suppression promotes alcoholic liver disease by inducing overgrowth of intestinal Enterococcus. Nat. Commun..

[169] Boo T., Cryan B., O’Donnell A., Fahy G. (2005). Prosthetic valve endocarditis caused by *Veillonella parvula*. J. Infect..

[170] Houston S., Taylor D., Rennie R. (1997). Prosthetic valve endocarditis due to *Veillonella dispar*: Successful medical treatment following penicillin desensitization. Clin. Infect. Dis..

[171] Loughrey A.C., Chew E.W. (1990). Endocarditis caused by *Veillonella dispar*. J. Infect..

[172] Marchandin H., Jean-Pierre H., Carrière C., Canovas F., Darbas H., Jumas-Bilak E. (2001). Prosthetic joint infection due to *Veillonella dispar*. Eur. J. Clin. Microbiol. Infect. Dis..

[173] Bhatti M.A., Frank M.O. (2000). *Veillonella parvula* Meningitis: Case Report and Review of *Veillonella* Infections. Clin. Infect. Dis..

[174] De Cruz P., Kang S., Wagner J., Buckley M., Sim W.H., Prideaux L. (2015). Association between specific mucosa-associated micro-biota in Crohn’s disease at the time of resection and subsequent disease recurrence: A pilot study. J. Gastroenterol. Hepatol..

[175] Horvath A., Rainer F., Bashir M., Leber B., Schmerboeck B., Klymiuk I., Groselj-Strele A., Durdevic M., Freedberg D.E., Abrams J.A. (2019). Biomarkers for oralization during long-term proton pump inhibitor therapy predict survival in cirrhosis. Sci. Rep..

[176] Voigtländer T., Leuchs E., Vonberg R.-P., Solbach P., Manns M.P., Suerbaum S., Lankisch T.O. (2015). Microbiological analysis of bile and its impact in critically ill patients with secondary sclerosing cholangitis. J. Infect..

[177] Wu L., Feng J., Li J., Yu Q., Ji J., Wu J., Dai W., Guo C. (2021). The gut microbiome-bile acid axis in hepatocarcinogenesis. Biomed. Pharmacother..

[178] Solé C., Guilly S., Da Silva K., Llopis M., Le-Chatelier E., Huelin P., Carol M., Moreira R., Fabrellas N., De Prada G. (2021). Alterations in Gut Microbiome in Cirrhosis as Assessed by Quantitative Metagenomics: Relationship with Acute-on-Chronic Liver Failure and Prognosis. Gastroenterology.

[179] Rocco A., Compare D., Angrisani D., Zamparelli M.S., Nardone G. (2014). Alcoholic disease: Liver and beyond. World J. Gastroenterol..

[180] Llopis M., Cassard A.M., Wrzosek L., Boschat L., Bruneau A., Ferrere G., Puchois V., Martin J.C., Lepage P., Le Roy T. (2016). Intestinal microbiota contributes to individual susceptibility to alcoholic liver disease. Gut.

[181] Gurwara S., Dai A., Ajami N.J., Graham D.Y., White D.L., Chen L., Jang A., Chen E., El-Serag H.B., Petrosino J.F. (2020). Alcohol use alters the colonic mucosa–associated gut microbiota in humans. Nutr. Res..

[182] Lebrun E.S., Nighot M., Dharmaprakash V., Kumar A., Lo C.-C., Chain P.S.G., Ma T.Y. (2020). The Gut Microbiome and Alcoholic Liver Disease: Ethanol Consumption Drives Consistent and Reproducible Alteration in Gut Microbiota in Mice. Life.

[183] Stadlbauer V., Horvath A., Komarova I., Schmerboeck B., Feldbacher N., Wurm S., Klymiuk I., Durdevic M., Rainer F., Blesl A. (2019). A single alcohol binge impacts on neu-trophil function without changes in gut barrier function and gut microbiome composition in healthy volunteers. PLoS ONE.

[184] Duan Y., Llorente C., Lang S., Brandl K., Chu H., Jiang L., White R.C., Clarke T.H., Nguyen K., Torralba M. (2019). Bacteriophage targeting of gut bacterium attenuates alcoholic liver disease. Nature.

[185] Lang S., Demir M., Duan Y., Martin A., Schnabl B. (2020). Cytolysin-positive *Enterococcus faecalis* is not increased in patients with non-alcoholic steatohepatitis. Liver Int..

[186] Vaga S., Lee S., Ji B., Andreasson A., Talley N.J., Agréus L., Bidkhori G., Kovatcheva-Datchary P., Park J., Lee D. (2020). Compositional and functional differences of the mucosal microbiota along the intestine of healthy individuals. Sci. Rep..

[187] Little R., Wine E., Kamath B.M., Griffiths A.M., Ricciuto A. (2020). Gut microbiome in primary sclerosing cholangitis: A review. World J. Gastroenterol..

[188] Sundin J., Aziz I., Nordlander S., Polster A., Hu Y.O.O., Hugerth L.W., Pennhag A., Engstrand L., Ohman L. (2020). Evidence of altered mucosa-associated and fecal microbiota composition in patients with Irritable Bowel Syndrome. Sci. Rep..

[189] Yang Q., Liang Q., Balakrishnan B., Belobrajdic D.P., Feng Q.-J., Zhang W. (2020). Role of Dietary Nutrients in the Modulation of Gut Microbiota: A Narrative Review. Nutrients.

[190] Pérez-Montes de Oca A., Julián M.T., Ramos A., Puig-Domingo M., Alonso N. (2020). Microbiota, Fiber, and NAFLD: Is There Any Connection?. Nutrients.

[191] Kummen M., Thingholm L.B., Rühlemann M.C., Holm K., Hansen S.H., Moitinho-Silva L., Liwinski T., Zenouzi R., Storm-Larsen C., Midttun Ø. (2020). Altered Gut Microbial Metabolism of Essential Nutrients in Primary Sclerosing Cholangitis. Gastroenterology.

[192] Lapidot Y., Amir A., Ben-Simon S., Veitsman E., Cohen-Ezra O., Davidov Y., Weiss P., Bradichevski T., Segev S., Koren O. (2021). Alterations of the salivary and fecal microbiome in patients with primary sclerosing cholangitis. Hepatol. Int..

[193] Quraishi M.N., Acharjee A., Beggs A.D., Horniblow R., Tselepis C., Gkoutos G. (2020). A Pilot Integrative Analysis of Colonic Gene Expression, Gut Microbiota, and Immune Infiltration in Primary Sclerosing Cholangitis-Inflammatory Bowel Disease: Association of Disease with Bile Acid Pathways. J. Crohns Colitis..

[194] Lemoinne S., Kemgang A., Ben Belkacem K., Straube M., Jegou S., Corpechot C., Chazouillères O., Housset C., Sokol H., Network S.-A.I. (2020). Fungi participate in the dysbiosis of gut microbiota in patients with primary sclerosing cholangitis. Gut.

[195] Rühlemann M., Liwinski T., Heinsen F.-A., Bang C., Zenouzi R., Kummen M., Thingholm L., Tempel M., Lieb W., Karlsen T. (2019). Consistent alterations in faecal microbiomes of patients with primary sclerosing cholangitis independent of associated colitis. Aliment. Pharmacol. Ther..

[196] Kummen M., Holm K., Anmarkrud J.A., Nygård S., Vesterhus M., Høivik M.L., Trøseid M., Marschall H.-U., Schrumpf E., Moum B. (2017). The gut microbial profile in patients with primary sclerosing cholangitis is distinct from patients with ulcerative colitis without biliary disease and healthy controls. Gut.

[197] Bajer L., Kverka M., Kostovcik M., Macinga P., Dvorak J., Stehlikova Z., Brezina J., Wohl P., Spicak J., Drastich P. (2017). Distinct gut microbiota profiles in patients with primary sclerosing cholangitis and ulcerative colitis. World J. Gastroenterol..

[198] Sabino J., Vieira-Silva S., Machiels K., Joossens M., Falony G., Ballet V., Ferrante M., Van Assche G., Van Der Merwe S., Vermeire S. (2016). Primary sclerosing cholangitis is characterised by intestinal dysbiosis independent from IBD. Gut.

[199] Torres J., Bao X., Goel A., Colombel J.-F., Pekow J., Jabri B., Williams K., Castillo A., Odin J., Meckel K. (2016). The features of mucosa-associated microbiota in primary sclerosing cholangitis. Aliment. Pharmacol. Ther..

[200] Kevans D., Tyler A.D., Holm K., Jørgensen K.K., Vatn M.H., Karlsen T.H., Kaplan G.G., Eksteen B. (2016). Characterization of Intestinal Microbiota in Ulcerative Colitis Patients with and without Primary Sclerosing Cholangitis. J. Crohns Colitis..

[201] Rossen N.G., Fuentes S., Boonstra K., D’Haens G.R., Heilig H.G., Zoetendal E.G., De Vos W.M., Ponsioen C.Y. (2015). The Mucosa-associated Microbiota of PSC Patients is Characterized by Low Diversity and Low Abundance of Uncultured Clostridiales II. J. Crohns Coliti.

[202] Turnbaugh P.J., Hamady M., Yatsunenko T., Cantarel B.L., Duncan A., Ley R.E. (2009). A core gut microbiome in obese and lean twins. Nature.

[203] Prosberg M., Bendtsen F., Vind I., Petersen A.M., Gluud L.L. (2016). The association between the gut microbiota and the inflammatory bowel disease activity: A systematic review and meta-analysis. Scand. J. Gastroenterol..

[204] Weinstein N., Garten B., Vainer J., Minaya D., Czaja K. (2020). Managing the Microbiome: How the Gut Influences Development and Disease. Nutrients.

[205] Allegretti J.R., Kassam Z., Carrellas M., Mullish B.H., Marchesi J.R., Pechlivanis A., Smith M., Gerardin Y., Timberlake S., Pratt D. (2019). Fecal Microbiota Transplantation in Patients With Primary Sclerosing Cholangitis: A Pilot Clinical Trial. Am. J. Gastroenterol..

[206] Wei Y., Li Y., Yan L., Sun C., Miao Q., Wang Q., Xiao X., Lian M., Li B., Chen Y. (2020). Alterations of gut microbiome in autoimmune hepatitis. Gut.

[207] Kim S.S., Eun J.W., Cho H.J., Song D.S., Kim C.W., Kim Y.S., Lee S.W., Kim Y.-K., Yang J., Choi J. (2020). Microbiome as a potential diagnostic and predictive biomarker in severe alcoholic hepatitis. Aliment. Pharmacol. Ther..

[208] Lang S., Fairfied B., Gao B., Duan Y., Zhang X., Fouts D.E., Schnabl B. (2020). Changes in the fecal bacterial microbiota associated with disease severity in alcoholic hepatitis patients. Gut Microbes.

[209] Tang Y., Zhou H., Xiang Y., Cui F. (2020). The diagnostic potential of gut microbiome for early hepatitis B virus-related hepatocellular carcinoma. Eur. J. Gastroenterol. Hepatol..

[210] Sung C.M., Lin Y.-F., Chen K.-F., Ke H.-M., Huang H.-Y., Gong Y.-N., Tsai W.-S., You J.-F., Lu M.J., Cheng H.-T. (2019). Predicting Clinical Outcomes of Cirrhosis Patients With Hepatic Encephalopathy From the Fecal Microbiome. Cell. Mol. Gastroenterol. Hepatol..

[211] Matera G., Muto V., Vinci M., Zicca E., Abdollahi-Roodsaz S., Van De Veerdonk F.L., Kullberg B.-J., Liberto M.C., Van Der Meer J.W.M., Focà A. (2009). Receptor Recognition of and Immune Intracellular Pathways for *Veillonella parvula* Lipopolysaccharide. Clin. Vaccine Immunol..

[212] Leylabadlo H.E., Ghotaslou R., Feizabadi M.M., Farajnia S., Moaddab S.Y., Ganbarov K., Khodadadi E., Tanomand A., Sheykhsaran E., Yousefi B. (2020). The critical role of *Faecalibacterium prausnitzii* in human health: An overview. Microb. Pathog..

[213] Tilg H., Cani P., Mayer E.A. (2016). Gut microbiome and liver diseases. Gut.

[214] Lin C.-Y., Lee A.H., Chiu K.K., Vieson M.D., Steelman A.J., Swanson K.S. (2020). *Saccharomyces cerevisiae* Fermentation Product Did Not Attenuate Clinical Signs, but Psyllium Husk Has Protective Effects in a Murine Dextran Sulfate Sodium-Induced Colitis Model. Curr. Dev. Nutr..

[215] Eeckhaut V., Machiels K., Perrier C., Romero C., Maes S., Flahou B., Steppe M., Haesebrouck F., Sas B. (2013). *Butyricicoccus pullicaecorum* in inflammatory bowel disease. Gut.

[216] Devriese S., Eeckhaut V., Geirnaert A., Bossche L.V.D., Hindryckx P., Van De Wiele T., Van Immerseel F., Ducatelle R., De Vos M., Laukens D. (2016). Reduced Mucosa-associated *Butyricicoccus* Activity in Patients with Ulcerative Colitis Correlates with Aberrant Claudin-1 Expression. J. Crohns Coliti.

[217] Zhang Q., Wu Y., Wang J., Wu G., Long W., Xue Z., Wang L., Zhang X., Pang X., Zhao Y. (2016). Accelerated dysbiosis of gut microbiota during aggravation of DSS-induced colitis by a butyrate-producing bacterium. Sci. Rep..

[218] Furukawa M., Moriya K., Nakayama J., Inoue T., Momoda R., Kawaratani H., Namisaki T., Sato S., Douhara A., Kaji K. (2020). Gut dysbiosis associated with clinical prognosis of patients with primary biliary cholangitis. Hepatol. Res..

[219] Abe K., Takahashi A., Fujita M., Imaizumi H., Hayashi M., Okai K., Ohira H. (2018). Dysbiosis of oral microbiota and its association with salivary immunological biomarkers in autoimmune liver disease. PLoS ONE.

[220] Tang R., Wei Y., Li Y., Chen W., Chen H., Wang Q., Yang F., Miao Q., Xiao X., Zhang H. (2018). Gut microbial profile is altered in primary biliary cholangitis and partially restored after UDCA therapy. Gut.

[221] Lv L.-X., Fang D.-Q., Shi D., Chen D.-Y., Yan R., Zhu Y.-X., Chen Y.-F., Shao L., Guo F.-F., Wu W.-R. (2016). Alterations and correlations of the gut microbiome, metabolism and immunity in patients with primary biliary cirrhosis. Environ. Microbiol..

[222] Joyce S.A., Gahan C.G. (2016). Bile Acid Modifications at the Microbe-Host Interface: Potential for Nutraceutical and Pharmaceutical Interventions in Host Health. Annu. Rev. Food Sci. Technol..

[223] Iliev I.D., Cadwell K. (2021). Effects of Intestinal Fungi and Viruses on Immune Responses and Inflammatory Bowel Diseases. Gastroenterology.

[224] Hallen-Adams H.E., Suhr M.J. (2017). Fungi in the healthy human gastrointestinal tract. Virulence.

[225] David L.A., Maurice C.F., Carmody R.N., Gootenberg D.B., Button J.E., Wolfe B.E., Ling A.V., Devlin A.S., Varma Y., Fischbach M.A. (2014). Diet rapidly and reproducibly alters the human gut microbiome. Nat. Cell Biol..

[226] Nagpal R., Neth B.J., Wang S., Mishra S.P., Craft S., Yadav H. (2020). Gut mycobiome and its interaction with diet, gut bacteria and alzheimer’s disease markers in subjects with mild cognitive impairment: A pilot study. EBioMedicine.

[227] Suhr M.J., Hallen-Adams H.E. (2015). The human gut mycobiome: Pitfalls and potential—A mycologist’s perspective. Mycologia.

[228] Gouba N., Raoult D., Drancourt M. (2013). Plant and fungal diversity in gut microbiota as revealed by molecular and culture investigations. PLoS ONE.

[229] Desnos-Ollivier M., Ragon M., Robert V., Raoux D., Gantier J.-C., Dromer F. (2008). Debaryomyces hansenii (*Candida famata*), a Rare Human Fungal Pathogen Often Misidentified as *Pichia guilliermondii* (*Candida guilliermondii*). J. Clin. Microbiol..

[230] Nash A.K., Auchtung T.A., Wong M.C., Smith D.P., Gesell J.R., Ross M.C., Stewart C.J., Metcalf G.A., Muzny D.M., Gibbs R.A. (2017). The gut mycobiome of the Human Microbiome Project healthy cohort. Microbiome.

[231] Hoffmann C., Dollive S., Grunberg S., Chen J., Li H., Wu G.D., Lewis J.D., Bushman F.D. (2013). Archaea and Fungi of the Human Gut Microbiome: Correlations with Diet and Bacterial Residents. PLoS ONE.

[232] Matijašić M., Meštrović T., Paljetak H., Perić M., Barešić A., Verbanac D. (2020). Gut Microbiota beyond Bacteria-Mycobiome, Virome, Archaeome, and Eukaryotic Parasites in IBD. Int. J. Mol. Sci..

[233] Vesty A., Biswas K., Taylor M.W., Gear K., Douglas R.G. (2017). Evaluating the Impact of DNA Extraction Method on the Representation of Human Oral Bacterial and Fungal Communities. PLoS ONE.

[234] Diaz P.I., Hong B.-Y., Dupuy A.K., Strausbaugh L.D. (2016). Mining the oral mycobiome: Methods, components, and meaning. Virulence.

[235] Jiang L., Stärkel P., Fan J.-G., Fouts D.E., Bacher P., Schnabl B. (2021). The gut mycobiome: A novel player in chronic liver diseases. J. Gastroenterol..

[236] Chin V.K., Yong V.C., Chong P.P., Nordin S.A., Basir R., Abdullah M. (2020). Mycobiome in the Gut: A Multiperspective Review. Mediat. Inflamm..

[237] Mason K.L., Downward J.R., Mason K.D., Falkowski N.R., Eaton K.A., Kao J.Y. (2012). *Candida albicans* and bacterial microbiota interactions in the cecum during recolonization following broad-spectrum antibiotic therapy. Infect. Immun..

[238] Qiu X., Zhang F., Yang X., Wu N., Jiang W., Li X., Li X., Liu Y. (2015). Changes in the composition of intestinal fungi and their role in mice with dextran sulfate sodium-induced colitis. Sci. Rep..

[239] Castagliuolo I., Riegler M.F., Valenick L., LaMont J.T., Pothoulakis C. (1999). *Saccharomyces boulardii* protease inhibits the effects of *Clostridium* difficile toxins A and B in human colonic mucosa. Infect. Immun..

[240] Buts J.-P., DeKeyser N., Stilmant C., Delem E., Smets F., Sokal E. (2006). *Saccharomyces boulardii* Produces in Rat Small Intestine a Novel Protein Phosphatase that Inhibits *Escherichia coli* Endotoxin by Dephosphorylation. Pediatr. Res..

[241] Brown G.D. (2005). Dectin-1: A signalling non-TLR pattern-recognition receptor. Nat. Rev. Immunol..

[242] Tang C., Kamiya T., Liu Y., Kadoki M., Kakuta S., Oshima K., Hattori M., Takeshita K., Kanai T., Saijo S. (2015). Inhibition of Dectin-1 Signaling Ameliorates Colitis by Inducing Lactobacillus-Mediated Regulatory T Cell Expansion in the Intestine. Cell Host Microbe.

[243] Iliev I.D., Funari V.A., Taylor K.D., Nguyen Q., Reyes C.N., Strom S.P., Brown J., Becker C.A., Fleshner P.R., Dubinsky M. (2012). Interactions Between Commensal Fungi and the C-Type Lectin Receptor Dectin-1 Influence Colitis. Science.

[244] Beheshti-Maal A., Shahrokh S., Ansari S., Mirsamadi E.S., Yadegar A., Mirjalali H., Zali M.R. (2021). Gut mycobiome: The probable determinative role of fungi in IBD patients. Mycoses.

[245] Qin J., Li R., Raes J., Arumugam M., Burgdorf K.S., Manichanh C., Nielsen T., Pons N., Levenez F., Yamada T. (2010). A human gut microbial gene catalogue established by metagenomic sequencing. Nature.

[246] Richard M.L., Sokol H. (2019). The gut mycobiota: Insights into analysis, environmental interactions and role in gastrointestinal diseases. Nat. Rev. Gastroenterol. Hepatol..

[247] Sokol H., Leducq V., Aschard H., Pham H.-P., Jegou S., Landman C., Cohen D., Liguori G., Bourrier A., Nion-Larmurier I. (2016). Fungal microbiota dysbiosis in IBD. Gut.

[248] Chin S.-F., Azlan P.I.H.M.M., Mazlan L., Neoh H.-M. (2018). Identification of *Schizosaccharomyces pombe* in the guts of healthy individuals and patients with colorectal cancer: Preliminary evidence from a gut microbiome secretome study. Gut Pathog..

[249] Luan C., Xie L., Yang X., Miao H., Lv N., Zhang R., Xiao X., Hu Y., Liu Y., Wu N. (2015). Dysbiosis of Fungal Microbiota in the Intestinal Mucosa of Patients with Colorectal Adenomas. Sci. Rep..

[250] Mar Rodríguez M., Pérez D., Javier Chaves F., Esteve E., Marin-Garcia P., Xifra G., Vendrell J., Jové M., Pamplona R., Ricart W. (2015). Obesity changes the human gut myco-biome. Sci. Rep..

[251] Chacón M., Lozano-Bartolomé J., Portero-Otín M., Rodríguez M., Xifra G., Puig J., Blasco G., Ricart W., Chaves F., Fernández-Real J. (2018). The gut mycobiome composition is linked to carotid atherosclerosis. Benef. Microbes.

[252] Zorena K., Kowalewska B., Szmigiero-Kawko M., Wąż P., Myśliwiec M. (2016). Higher diversity in fungal species discriminates children with type 1 diabetes mellitus from healthy control. Patient Prefer. Adherence.

[253] Das A., O’Herlihy E., Shanahan F., O’Toole P.W., Jeffery I.B. (2021). The fecal mycobiome in patients with Irritable Bowel Syndrome. Sci. Rep..

[254] Botschuijver S., Roeselers G., Levin E., Jonkers D.M., Welting O., Heinsbroek S.E., de Weerd H.H., Boekhout T., Fornai M., Masclee A.A. (2017). Intestinal Fungal Dysbiosis Is Associated With Visceral Hypersensitivity in Patients With Irritable Bowel Syndrome and Rats. Gastroenterology.

[255] Sivignon A., de Vallée A., Barnich N., Denizot J., Darcha C., Pignède G. (2015). *Saccharomyces cerevisiae* CNCM I-3856 prevents colitis induced by AIEC bacteria in the transgenic mouse model mimicking Crohn’s disease. Inflamm. Bowel Dis..

[256] Rühlemann M.C., Solovjeva M.E.L., Zenouzi R., Liwinski T., Kummen M., Lieb W., Hov J.R., Schramm C., Franke A., Bang C. (2020). Gut mycobiome of primary sclerosing cholangitis patients is characterised by an increase of *Trichocladium griseum* and *Candida* species. Gut.

[257] Katt J., Schwinge D., Schoknecht T., Quaas A., Sobottka I., Burandt E., Becker C., Neurath M.F., Lohse A.W., Herkel J. (2013). Increased T helper type 17 response to pathogen stimulation in patients with primary sclerosing cholangitis. Hepatology.

[258] Kagami S., Rizzo H.L., Kurtz S.E., Miller L.S., Blauvelt A. (2010). IL-23 and IL-17A, but Not IL-12 and IL-22, Are Required for Optimal Skin Host Defense against *Candida albicans*. J. Immunol..

[259] Chen K., McAleer J.P., Lin Y., Paterson D.L., Zheng M., Alcorn J.F., Weaver C.T., Kolls J.K. (2011). Th17 Cells Mediate Clade-Specific, Serotype-Independent Mucosal Immunity. Immunity.

[260] Littman D.R., Rudensky A.Y. (2010). Th17 and Regulatory T Cells in Mediating and Restraining Inflammation. Cell.

[261] Fujino S., Andoh A., Bamba S., Ogawa A., Hata K., Araki Y., Bamba T., Fujiyama Y. (2003). Increased expression of interleukin 17 in inflammatory bowel disease. Gut.

[262] Miossec P., Korn T., Kuchroo V.K. (2009). Interleukin-17 and Type 17 Helper T Cells. N. Engl. J. Med..

[263] Kulaksiz H., Rudolph G., Kloeters-Plachky P., Sauer P., Geiss H., Stiehl A. (2006). Biliary candida infections in primary sclerosing chol-angitis. J. Hepatol..

[264] Oztas E., Odemis B., Kekilli M., Kurt M., Dinc B.M., Parlak E., Kalkanci A., Sasmaz N. (2009). Systemic phaeohyphomycosis resembling primary sclerosing cholangitis caused by Exophiala dermatitidis. J. Med. Microbiol..

[265] Hong K.H., Kim J.W., Jang S.J., Yu E., Kim E.-C. (2009). Liver cirrhosis caused by Exophiala dermatitidis. J. Med. Microbiol..

[266] Kim M.-S., Park E.-J., Roh S.W., Bae J.-W. (2011). Diversity and Abundance of Single-Stranded DNA Viruses in Human Feces. Appl. Environ. Microbiol..

[267] Sweere J.M., Van Belleghem J.D., Ishak H., Bach M.S., Popescu M., Sunkari V., Kaber G., Manasherob R., Suh G.A., Cao X. (2019). Bacteriophage trigger antiviral immunity and prevent clearance of bacterial infection. Science.

[268] Guerin E., Hill C. (2020). Shining Light on Human Gut Bacteriophages. Front. Cell. Infect. Microbiol..

[269] Massimino L., Lovisa S., Lamparelli L.A., Danese S., Ungaro F. (2021). Gut eukaryotic virome in colorectal carcinogenesis: Is that a trigger?. Comput. Struct. Biotechnol. J..

[270] Manrique P., Bolduc B., Walk S.T., Van Der Oost J., De Vos W.M., Young M.J. (2016). Healthy human gut phageome. Proc. Natl. Acad. Sci. USA.

[271] Ungaro F., Massimino L., D’Alessio S., Danese S. (2019). The gut virome in inflammatory bowel disease pathogenesis: From meta-genomics to novel therapeutic approaches. United Eur. Gastroenterol. J..

[272] Ungaro F., Massimino L., Furfaro F., Rimoldi V., Peyrin-Biroulet L., D’Alessio S., Danese S. (2019). Metagenomic analysis of intestinal mucosa revealed a specific eukaryotic gut virome signature in early-diagnosed inflammatory bowel disease. Gut Microbes.

[273] Lim E.S., Zhou Y., Zhao G., Bauer I.K., Droit L., Ndao I.M. (2015). Early life dynamics of the human gut virome and bacterial microbiome in infants. Nat. Med..

[274] Dahlman S., Avellaneda-Franco L., Barr J.J. (2021). Phages to shape the gut microbiota?. Curr. Opin. Biotechnol..

[275] Zhang T., Breitbart M., Lee W.H., Run J.-Q., Wei C.L., Soh S.W.L., Hibberd M.L., Liu E.T., Rohwer F., Ruan Y. (2005). RNA Viral Community in Human Feces: Prevalence of Plant Pathogenic Viruses. PLoS Biol..

[276] Holtz L.R. (2020). Putting the Virome on the Map: The Influence of Host Geography and Ethnicity on the Gut Virome. Cell Host Microbe.

[277] Garmaeva S., Sinha T., Kurilshikov A., Fu J., Wijmenga C., Zhernakova A. (2019). Studying the gut virome in the metagenomic era: Challenges and perspectives. BMC Biol..

[278] Shkoporov A.N., Clooney A.G., Sutton T.D., Ryan F.J., Daly K.M., Nolan J.A., McDonnell S.A., Khokhlova E.V., Draper L.A., Forde A. (2019). The Human Gut Virome Is Highly Diverse, Stable, and Individual Specific. Cell Host Microbe.

[279] Gregory A.C., Zablocki O., Zayed A.A., Howell A., Bolduc B., Sullivan M.B. (2020). The Gut Virome Database Reveals Age-Dependent Patterns of Virome Diversity in the Human Gut. Cell Host Microbe.

[280] Zuo T., Sun Y., Wan Y., Yeoh Y.K., Zhang F., Cheung C.P., Chen N., Luo J., Wang W., Sung J.J. (2020). Human-Gut-DNA Virome Variations across Geography, Ethnicity, and Urbanization. Cell Host Microbe.

[281] Minot S., Sinha R., Chen J., Li H., Keilbaugh S.A., Wu G.D., Lewis J.D., Bushman F.D. (2011). The human gut virome: Inter-individual variation and dynamic response to diet. Genome Res..

[282] Gogokhia L., Buhrke K., Bell R., Hoffman B., Brown D.G., Hanke-Gogokhia C., Ajami N.J., Wong M.C., Ghazaryan A., Valentine J.F. (2019). Expansion of Bacteriophages Is Linked to Aggravated Intestinal Inflammation and Colitis. Cell Host Microbe.

[283] Lindfors K., Lin J., Lee H.-S., Hyöty H., Nykter M., Kurppa K., Liu E., Koletzko S., Rewers M., Hagopian W. (2020). Metagenomics of the faecal virome indicate a cumulative effect of enterovirus and gluten amount on the risk of coeliac disease autoimmunity in genetically at risk children: The TEDDY study. Gut.

[284] Nyström N., Berg T., Lundin E., Skog O., Hansson I., Frisk G., Juko-Pecirep I., Nilsson M., Gyllensten U., Finkel Y. (2013). Human Enterovirus Species B in Ileocecal Crohn’s Disease. Clin. Transl. Gastroenterol..

[285] Norman J.M., Handley S.A., Baldridge M.T., Droit L., Liu C.Y., Keller B.C., Kambal A., Monaco C.L., Zhao G., Fleshner P. (2015). Disease-Specific Alterations in the Enteric Virome in Inflammatory Bowel Disease. Cell.

[286] Nakatsu G., Zhou H., Wu W.K.K., Wong S.H., Coker O.O., Dai Z., Li X., Szeto C.-H., Sugimura N., Lam T.Y.-T. (2018). Alterations in Enteric Virome Are Associated With Colorectal Cancer and Survival Outcomes. Gastroenterology.

[287] Legoff J., Resche-Rigon M., Bouquet J., Robin M., Naccache S.N., Mercier-Delarue S. (2017). The eukaryotic gut virome in hematopoietic stem cell transplantation: New clues in enteric graft-versus-host disease. Nat. Med..

[288] Monaco C.L., Gootenberg D.B., Zhao G., Handley S.A., Ghebremichael M.S., Lim E.S., Lankowski A., Baldridge M.T., Wilen C.B., Flagg M. (2016). Altered Virome and Bacterial Microbiome in Human Immunodeficiency Virus-Associated Acquired Immunodeficiency Syndrome. Cell Host Microbe.

[289] Reyes A., Blanton L.V., Cao S., Zhao G., Manary M.J., Trehan I., Smith M.I., Wang D., Virgin H.W., Rohwer F. (2015). Gut DNA viromes of Malawian twins discordant for severe acute malnutrition. Proc. Natl. Acad. Sci. USA.

[290] Ma Y., You X., Mai G., Tokuyasu T., Liu C. (2018). A human gut phage catalog correlates the gut phageome with type 2 diabetes. Microbiome.

[291] Wook K., Allen D.W., Briese T., Couper J.J., Barry S.C., Colman P.G., Cotterill A.M., Davis E.A., Giles L.C., Harrison L.C. (2019). Distinct Gut Virome Profile of Pregnant Women With Type 1 Diabetes in the ENDIA Study. Open Forum Infect. Dis..

[292] Zuo T., Lu X.-J., Zhang Y., Cheung C.P., Lam S., Zhang F., Tang W., Ching J.Y.L., Zhao R., Chan P.K.S. (2019). Gut mucosal virome alterations in ulcerative colitis. Gut.

[293] Yan A., Butcher J., Mack D., Stintzi A. (2020). Virome Sequencing of the Human Intestinal Mucosal–Luminal Interface. Front. Cell. Infect. Microbiol..

[294] Zuo T., Wong S.H., Lam K., Lui R., Cheung K., Tang W., Ching Y.L., Chan P.C. (2018). Bacteriophage transfer during faecal microbiota transplantation in *Clostridium difficile* infection is associated with treatment outcome. Gut.

[295] Park H., Laffin M.R., Jovel J., Millan B., Hyun J.E., Hotte N., Kao D., Madsen K.L. (2019). The success of fecal microbial transplantation in Clostridium difficile infection correlates with bacteriophage relative abundance in the donor: A retrospective cohort study. Gut Microbes.

[296] Zhang F., Zuo T., Yeoh Y.K., Cheng F.W.T., Liu Q., Tang W., Cheung K.C.Y., Yang K., Cheung C.P., Mo C.C. (2021). Longitudinal dynamics of gut bacteriome, mycobiome and virome after fecal microbiota transplantation in graft-versus-host disease. Nat. Commun..

[297] Broecker F., Klumpp J., Moelling K. (2016). Long-term microbiota and virome in a Zürich patient after fecal transplantation against *Clostridium difficile* infection. Ann. N. Y. Acad. Sci..

[298] Draper L.A., Ryan F.J., Smith M.K., Jalanka J., Mattila E., Arkkila P.A., Ross R.P., Satokari R., Hill C. (2018). Long-term colonisation with donor bacteriophages following successful faecal microbial transplantation. Microbiome.

[299] Ott S.J., Waetzig G.H., Rehman A., Moltzau-Anderson J., Bharti R., Grasis J.A., Cassidy L., Tholey A., Fickenscher H., Seegert D. (2017). Efficacy of Sterile Fecal Filtrate Transfer for Treating Patients With Clostridium difficile Infection. Gastroenterology.

[300] Lang S., Demir M., Martin A., Jiang L., Zhang X., Duan Y., Gao B., Kasper P., Tacke F., Goeser T. (2020). Intestinal Virome Signature Associated with Severity of Nonalcoholic Fatty Liver Disease. Gastroenterology.

[301] Jiang L., Lang S., Duan Y., Zhang X., Gao B., Chopyk J., Schwanemann L.K., Ventura-Cots M., Bataller R., Bosques-Padilla F. (2020). Intestinal Virome in Patients with Alcoholic Hepatitis. Hepatology.

[302] Bajaj J.S., Sikaroodi M., Shamsaddini A., Henseler Z., Santiago-Rodriguez T., Acharya C., Fagan A., Hylemon P.B., Fuchs M., Gavis E. (2020). Interaction of bacterial metagenome and virome in patients with cirrhosis and hepatic encephalopathy. Gut.

[303] Thijssen M., Tacke F., Beller L., Deboutte W., Yinda K.C., Nevens F., Laleman W., Van Ranst M., Pourkarim M.R. (2020). Clinical relevance of plasma virome dynamics in liver transplant recipients. EBioMedicine.

[304] Letexier D., Diraison F., Beylot M. (2003). Addition of inulin to a moderately high-carbohydrate diet reduces hepatic lipogenesis and plasma triacylglycerol concentrations in humans. Am. J. Clin. Nutr..

[305] Ferrere G., Wrzosek L., Cailleux F., Turpin W., Puchois V., Spatz M., Ciocan D., Rainteau D., Humbert L., Hugot C. (2017). Fecal microbiota manipulation prevents dysbiosis and alcohol-induced liver injury in mice. J. Hepatol..

[306] Gibson G.R., Hutkins R., Sanders M.E., Prescott S.L., Reimer R.A., Salminen S.J., Scott K., Stanton C., Swanson K.S., Cani P.D. (2017). Expert consensus document: The International Scientific Association for Probiotics and Prebiotics (ISAPP) consensus statement on the definition and scope of prebiotics. Nat. Rev. Gastroenterol. Hepatol..

[307] Adachi Y., Bradford B.U., Gao W., Bojes H.K., Thurman R.G. (1994). Inactivation of Kupffer cells prevents early alcohol-induced liver injury. Hepatology.

[308] Horvath A., Durdevic M., Leber B., di Vora K., Rainer F., Krones E., Douschan P., Spindelboeck W., Durchschein F., Zollner G. (2020). Changes in the Intestinal Microbiome during a Multispecies Probiotic Intervention in Compensated Cirrhosis. Nutrients.

[309] Horvath A., Leber B., Schmerboeck B., Tawdrous M., Zettel G., Hartl A., Madl T., Stryeck S., Fuchs D., Lemesch S. (2016). Randomised clinical trial: The effects of a multispecies probiotic vs. placebo on innate immune function, bacterial translocation and gut permeability in patients with cirrhosis. Aliment. Pharmacol. Ther..

[310] Chi X., Pan C.Q., Liu S., Cheng D., Cao Z., Xing H. (2020). Regulating Intestinal Microbiota in the Prevention and Treatment of Alcohol-Related Liver Disease. Can. J. Gastroenterol. Hepatol..

[311] Han S.H., Suk K.T., Kim D.J., Kim M.Y., Baik S.K., Kim Y.D., Cheo G.J., Choi S.H., Ham L. (2015). Effects of probiotics (cultured *Lactobacillus subtilis*/*Streptococcus faecium*) in the treatment of alcoholic hepatitis: Randomized-controlled multicenter study. Eur. J. Gastroenterol. Hepatol..

[312] Stadlbauer V., Mookerjee R.P., Hodges S., Wright G.A., Davies N.A., Jalan R. (2008). Effect of probiotic treatment on deranged neutrophil function and cytokine responses in patients with compensated alcoholic cirrhosis. J. Hepatol..

[313] Cui Y., Qi S., Zhang W., Mao J., Tang R., Wang C., Liu J., Luo X.M., Wang H. (2019). *Lactobacillus reuteri* ZJ617 Culture Supernatant Attenuates Acute Liver Injury Induced in Mice by *Lipopolysaccharide*. J. Nutr..

[314] Ritze Y., Bárdos G., Claus A., Ehrmann V., Bergheim I., Schwiertz A., Bischoff S.C. (2014). *Lactobacillus rhamnosus* GG Protects against Non-Alcoholic Fatty Liver Disease in Mice. PLoS ONE.

[315] Bajaj J.S., Heuman D.M., Sanyal A.J., Hylemon P.B., Sterling R.K., Stravitz R.T., Fuchs M., Ridlon J.M., Daita K., Monteith P. (2013). Modulation of the Metabiome by Rifaximin in Patients with Cirrhosis and Minimal Hepatic Encephalopathy. PLoS ONE.

[316] Caraceni P., Vargas V., Solà E., Alessandria C., de Wit K., Trebicka J., Angeli P., Mookerjee R.P., Durand F., Pose E. (2021). The use of Rifaximin in Patients with Cirrhosis. Hepatology.

[317] Liu Y., Fan L., Cheng Z., Yu L., Cong S., Hu Y., Zhu L., Zhang B., Cheng Y., Zhao P. (2021). Fecal transplantation alleviates acute liver injury in mice through regulating Treg/Th17 cytokines balance. Sci. Rep..

[318] Philips C.A., Pande A., Shasthry S.M., Jamwal K.D., Khillan V., Chandel S.S., Kumar G., Sharma M.K., Maiwall R., Jindal A. (2017). Healthy Donor Fecal Microbiota Transplantation in Steroid-Ineligible Severe Alcoholic Hepatitis: A Pilot Study. Clin. Gastroenterol. Hepatol..

[319] Witjes J.J., Smits L.P., Pekmez C.T., Prodan A., Meijnikman A.S., Troelstra M.A., Bouter K.E., Herrema H., Levin E., Holleboom A.G. (2020). Donor Fecal Microbiota Transplantation Alters Gut Microbiota and Metabolites in Obese Individuals with Steatohepatitis. Hepatol. Commun..

[320] Safari Z., Monnoye M., Abuja P.M., Mariadassou M., Kashofer K., Gérard P., Zatloukal K. (2019). Steatosis and gut microbiota dysbiosis induced by high-fat diet are reversed by 1-week chow diet administration. Nutr. Res..

[321] Sheng L., Jena P.K., Hu Y., Liu H.X., Nagar N., Kalanetra K.M., French S.W., Mills D.A., Wan Y.-J.Y. (2017). Hepatic inflammation caused by dysregulated bile acid synthesis is reversible by butyrate supplementation. J. Pathol..

[322] Damms-Machado A., Louis S., Schnitzer A., Volynets V., Rings A., Basrai M., Bischoff S.C. (2017). Gut permeability is related to body weight, fatty liver disease, and insulin resistance in obese individuals undergoing weight reduction. Am. J. Clin. Nutr..

[323] Biolato M., Manca F., Marrone G., Cefalo C., Racco S., A Miggiano G., Valenza V., Gasbarrini A., Miele L., Grieco A. (2019). Intestinal permeability after Mediterranean diet and low-fat diet in non-alcoholic fatty liver disease. World J. Gastroenterol..

[324] Cao Y., Xiao Y., Zhou K., Yan J., Wang P., Yan W., Cai W. (2019). FXR agonist GW4064 improves liver and intestinal pathology and alters bile acid metabolism in rats undergoing small intestinal resection. Am. J. Physiol. Liver Physiol..

[325] Hsu B.B., Gibson T.E., Yeliseyev V., Liu Q., Lyon L., Bry L., Silver P.A., Gerber G.K. (2019). Dynamic Modulation of the Gut Microbiota and Metabolome by Bacteriophages in a Mouse Model. Cell Host Microbe.

[326] Szabo G. (2018). Gut-Liver Axis Beyond the Microbiome: How the Fungal Mycobiome Contributes to Alcoholic Liver Disease. Hepatology.

[327] Shah A., Crawford D., Burger D., Martin N., Walker M., Talley N.J., Tallis C., Jones M., Stuart K., Keely S. (2019). Effects of Antibiotic Therapy in Primary Sclerosing Cholangitis with and without Inflammatory Bowel Disease: A Systematic Review and Meta-Analysis. Semin. Liver Dis..

[328] Tabibian J.H., Weeding E., Jorgensen R.A., Petz J.L., Keach J.C., Talwalkar J.A., Lindor K.D. (2013). Randomised clinical trial: Vancomycin or metronidazole in patients with primary sclerosing cholangitis—A pilot study. Aliment. Pharmacol. Ther..

[329] Davies Y.K., Cox K.M., Abdullah B.A., Safta A., Terry A.B., Cox K.L. (2008). Long-term Treatment of Primary Sclerosing Cholangitis in Children with Oral Vancomycin: An Immunomodulating Antibiotic. J. Pediatr. Gastroenterol. Nutr..

[330] Howden B.P., Smith D.J., Mansell A., Johnson P.D.R., Ward P.B., Stinear T.P., Davies J.K. (2008). Different bacterial gene expression patterns and attenuated host immune responses are associated with the evolution of low-level vancomycin resistance during persistent methicillin-resistant *Staphylococcus aureus* bacteraemia. BMC Microbiol..

[331] Tabibian J.H., Gossard A., El-Youssef M., Eaton J.E., Petz J., Jorgensen R., Enders F.B., Tabibian A., Lindor K.D. (2017). Prospective Clinical Trial of Rifaximin Therapy for Patients With Primary Sclerosing Cholangitis. Am. J. Ther..

[332] Silveira M.G., Torok N.J., Gossard A.A., Keach J.C., Jorgensen R.R., Petz R.J.L., Lindor K.D. (2009). Minocycline in the Treatment of Patients with Primary Sclerosing Cholangitis: Results of a Pilot Study. Am. J. Gastroenterol..

[333] Vleggaar F.P., Monkelbaan J.F., Van Erpecum K.J. (2008). Probiotics in primary sclerosing cholangitis: A randomized placebo-controlled crossover pilot study. Eur. J. Gastroenterol. Hepatol..

[334] Shimizu M., Iwasaki H., Mase S., Yachie A. (2012). Successful Treatment of Primary Sclerosing Cholangitis with a Steroid and a Probiotic. Case Rep. Gastroenterol..

[335] Suri J., Patwardhan V., Bonder A. (2019). Pharmacologic management of primary sclerosing cholangitis: What’s in the pipeline?. Expert Rev. Gastroenterol. Hepatol..

[336] Kowdley K.V., Vuppalanchi R., Levy C., Floreani A., Andreone P., LaRusso N.F., Shrestha R., Trotter J., Goldberg D., Rushbrook S. (2020). A randomized, placebo-controlled, phase II study of obeticholic acid for primary sclerosing cholangitis. J. Hepatol..

[337] Fickert P., Hirschfield G.M., Denk G., Marschall H.-U., Altorjay I., Färkkilä M., Schramm C., Spengler U., Chapman R., Bergquist A. (2017). norUrsodeoxycholic acid improves cholestasis in primary sclerosing cholangitis. J. Hepatol..

[338] Cai S.-Y., He H., Nguyen T., Mennone A., Boyer J.L. (2010). Retinoic acid represses CYP7A1 expression in human hepatocytes and HepG2 cells by FXR/RXR-dependent and independent mechanisms. J. Lipid Res..

[339] Hirschfield G.M., Chazouillères O., Drenth J.P., Thorburn D., Harrison S.A., Landis C.S., Mayo M.J., Muir A.J., Trotter J.F., Leeming D.J. (2019). Effect of NGM282, an FGF19 analogue, in primary sclerosing cholangitis: A multicenter, randomized, double-blind, placebo-controlled phase II trial. J. Hepatol..

[340] Trauner M., Gulamhusein A., Hameed B., Caldwell S., Shiffman M.L., Landis C., Eksteen B., Agarwal K., Muir A., Rushbrook S. (2019). The Nonsteroidal Farnesoid X Receptor Agonist Cilofexor (GS-9674) Improves Markers of Cholestasis and Liver Injury in Patients with Primary Sclerosing Cholangitis. Hepatology.

[341] Prokopič M., Beuers U. (2021). Management of primary sclerosing cholangitis and its complications: An algorithmic approach. Hepatol. Int..

[342] Yokoda R.T., Rodriguez E.A. (2020). Review: Pathogenesis of cholestatic liver diseases. World J. Hepatol..

[343] European Association for the Study of the Liver (2017). EASL Clinical Practice Guidelines: The diagnosis and management of patients with primary biliary cholangitis. J. Hepatol..

